# High-definition Cathodal Direct Current Stimulation for Treatment of Acute Ischemic Stroke: A Randomized Clinical Trial

**DOI:** 10.1001/jamanetworkopen.2023.19231

**Published:** 2023-06-21

**Authors:** Mersedeh Bahr-Hosseini, Kambiz Nael, Gozde Unal, Marco Iacoboni, David S. Liebeskind, Marom Bikson, Jeffrey L. Saver

**Affiliations:** 1 Department of Neurology and Comprehensive Stroke Center, David Geffen School of Medicine at UCLA, Los Angeles, California; 2 Department of Radiology, David Geffen School of Medicine at UCLA, Los Angeles, California; 3 Department of Biomedical Engineering, The City College of New York (CCNY), New York; 4 Department of Psychiatry and Biobehavioral Sciences, David Geffen School of Medicine at UCLA, Los Angeles, California

## Abstract

**Importance:**

Cathodal transcranial direct current stimulation (C-tDCS) provides neuroprotection in preclinical models of acute ischemic stroke (AIS) by inhibiting peri-infarct excitotoxic effects and enhancing collateral perfusion due to its vasodilatory properties.

**Objective:**

To report the first-in-human pilot study using individualized high-definition (HD) C-tDCS as a treatment of AIS.

**Design, Setting, and Participants:**

This randomized clinical trial was sham controlled with 3 + 3 dose escalation design, and was conducted at a single center from October 2018 to July 2021. Eligible participants were treated for AIS within 24 hours from onset, had imaging evidence of cortical ischemia with salvageable penumbra, and were ineligible for reperfusion therapies. HD C-tDCS electrode montage was selected for each patient to deliver the electric current to the ischemic region only. Patients were followed for 90 days.

**Main Outcomes and Measures:**

Primary outcomes were feasibility, assessed as time from randomization to study stimulation initiation; tolerability, assessed by rate of patients completing the full study stimulation period; and safety, assessed by rates of symptomatic intracranial hemorrhage at 24 hours. The efficacy imaging biomarkers of neuroprotection and collateral enhancement were explored.

**Results:**

A total of 10 patients with AIS were enrolled, 7 were randomized to active treatment and 3 to sham. Patient age was mean (SD) 75 (10) years old, 6 (60%) were female, and National Institutes of Health Stroke Scale score was mean (SD) 8 (7). Two doses of HD C-tDCS (1 milliamp [mA] for 20 minutes and 2 mA for 20 minutes) were studied. The speed of HD C-tDCS implementation was a median (IQR) 12.5 minutes (9-15 minutes) in the last 4 patients. Patients tolerated the HD C-tDCS with no permanent stimulation cessation. The hypoperfused region was reduced by a median (IQR) 100% (46% to 100%) in the active group vs increased by 325% (112% to 412%) in sham. Change in quantitative relative cerebral blood volume early poststimulation was a median (IQR) 64% (40% to 110%) in active vs −4% (−7% to 1%) sham patients and followed a dose-response pattern. Penumbral salvage in the active C-tDCS group was median (IQR) 66% (29% to 80.5%) vs 0% (IQR 0% to 0%) in sham.

**Conclusion and Relevance:**

In this randomized, first-in-human clinical trial, HD C-tDCS was started efficiently and well tolerated in emergency settings, with signals of beneficial effect upon penumbral salvage. These results support advancing HD C-tDCS to larger trials.

**Trial Registration:**

ClinicalTrials.gov Identifier: NCT03574038

## Key Points

**Question:**

Is application of high-definition cathodal transcranial direct current stimulation (HD C-tDCS) as a noninvasive targeted acute ischemic stroke treatment strategy feasible and well-tolerated, and does it show signals of beneficial effects?

**Findings:**

In this randomized clinical trial enrolling 10 patients (7 active, 3 sham), HD C-tDCS was started within a median 12.5 minutes of randomization in final enrolled patients and showed good tolerability with signals of favorable effects on salvage of threatened tissue.

**Meaning:**

These results suggest that HD C-tDCS is a noninvasive targeted acute ischemic stroke treatment strategy that can be efficiently applied in emergency settings and warrants testing in larger multicenter trials.

## Teaser

This randomized clinical trial examines the efficacy and feasibility of high-definition cathodal transcranial direct current stimulation for treatment of acute ischemic stroke.

## Introduction

Acute ischemic stroke (AIS) is a leading cause of death and disability across the US and around the world.^1^ Treatments for AIS are currently limited to reperfusion therapies: intravenous thrombolysis and endovascular thrombectomy. Many patients are not candidates for these therapies, and among those who receive them, the rate of excellent outcome remains low; only 20% to 30% are free from disability at 3 months poststroke.^2,3,4^ Therefore, there is a critical need to develop additional therapies for patients with AIS. In animal models of acute cerebral ischemia, cathodal transcranial direct current stimulation (C-tDCS) salvages ischemic tissue at risk of infarction, both through direct neuroprotection by inhibiting peri-infarct excitotoxic effects, inflammatory and apoptotic pathways,^5^ and through collateral perfusion enhancement by inducing vasodilation.^6^ C-tDCS has many advantages over existing therapies. Delivery of pharmacological agents to threatened tissues is constrained by the same blood flow diminution that is producing ischemia. In contrast, C-tDCS is a regionally directed therapy that instantly reaches maximum local concentration.^7^ In addition, via high-definition (HD) electrode montages, electrical field shape and coverage location can be tailored to the ischemic tissue at risk of infarction.

We performed a first-in-human pilot study of HD C-tDCS as an AIS treatment strategy in patients with acute cortical ischemic strokes who harbored salvageable penumbra. We report the results of our proof-of-concept study, which was designed to assess the feasibility, safety, and tolerability of applying individualized HD C-tDCS in acute stroke emergency settings and explore potential efficacy on imaging biomarkers of neuroprotection and collateral enhancement.

## Methods

The study design was a randomized (3 active: 1 sham), sham-controlled, 3 + 3 dose-escalation clinical trial (protocol available in Supplement 1). Key inclusion criteria were: within 24 hours from AIS onset; imaging evidence of cortical ischemia; presence of salvageable penumbra; and ineligibility for reperfusion therapies (intravenous lytics and endovascular thrombectomy) (eTable 1 in Supplement 2). Patients who met the entry criteria were enrolled after obtaining informed consent from patients or their legally authorized representative. The study was performed under a US Food and Drug Administration (FDA) Investigational Device Exemption for a Soterix Medical HD tDCS device. The study protocol was reviewed and approved by the University of California, Los Angeles (UCLA) local institutional review board. This study report follows the Consolidated Standards of Reporting Trials (CONSORT) reporting guideline for randomized clinical trials.

A total of 6 dose tiers were initially planned, increasing in intensity or duration of stimulation. The occurrence of symptomatic intracranial hemorrhage (SICH) determined the escalation pace through the tiers (eMethods in Supplement 2).

**Figure 1.**
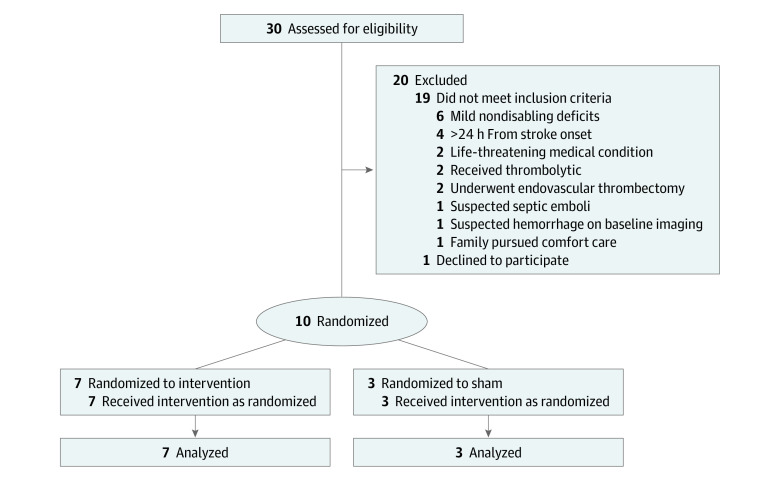
Study Flow Diagram

HD C-tDCS was delivered to the ischemic tissue using individualized montages. Six HD montages were predesigned to cover 6 vascular distribution-specific ischemic fields caused by occlusions of the middle cerebral artery (MCA) trunk, MCA superior division, MCA inferior division, posterior cerebral artery, anterior cerebral artery, and posterior inferior cerebellar artery. The coverage of ischemic regions by electric fields was estimated via computational modeling of the electrical current flow (Figure 1).

The time from randomization to study stimulation initiation was recorded for the feasibility outcome. Tolerability was assessed with 2 end points: (1) the rate of patients completing the full study stimulation period (primary tolerability end point); and (2) a formal tolerability technician questionnaire (secondary tolerability end point). Immediately following the stimulation cycle, the technician performed a visual inspection of the skin and completed the tolerability form. The tolerability form consisted of 14 validated cutaneous, neurological, and pain items from the Patient-Reported Outcomes version of the Common Terminology Criteria for Adverse Events (PRO-CTAE).^8^ The skin was inspected after each stimulation for presence of rash or skin discoloration and patients were queried on cutaneous symptoms such as pain, numbness, and tingling, and new neurological symptoms including dizziness or headache. Each item was scored as absent or present and subsequently graded for severity (mild, moderate, or severe), its effect on the stimulation protocol, the symptom’s duration, and whether any intervention was done to remedy the symptoms (form available with study protocol in Supplement 2).

The imaging biomarkers of neuroprotection and collateral enhancement were characterized at 2 to 4 hours (early time point) and 24 to 30 hours (late time point) and included improved perfusion (reduction in hypoperfusion region volume); collateral enhancement (increase in quantified relative cerebral blood volume [qrCBV]); and penumbral tissue salvage (tissue at risk not progressing to infarction). The modified Rankin Scale (mRS) was measured at 90 days for exploratory clinical efficacy analysis.

### Statistical Analysis

The demographic and baseline clinical characteristics of the study population were delineated with standard descriptive statistics. Categorical variables were summarized by frequencies. Continuous variables were characterized by means and standard deviations or median values with interquartile ranges (IQRs). Due to the descriptive nature of the primary feasibility and tolerability end points, tests of statistical significance were not performed.

## Results

**Figure 2.**
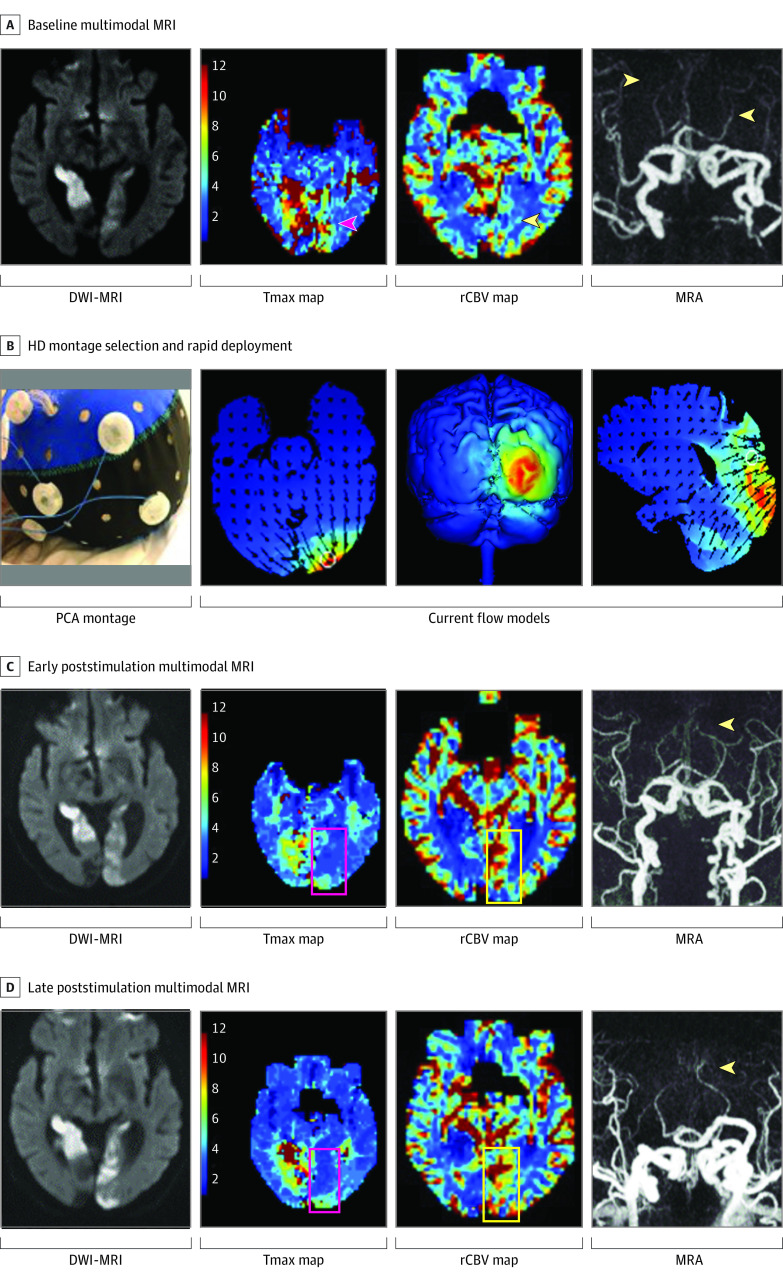
First Exemplar of a Transcranial Electrical Stimulation in Stroke Early After Onset Clinical Trial Patient Patient 1 was a male in his 70s with acute ischemic stroke due to bilateral posterior cerebral artery (PCA) occlusions. Given the symptomatic nature of the left PCA occlusion, he received 20 minutes of 1 milliamp (mA) high-definition (HD) cathodal transcranial direct current stimulation to the left occipital region only. Early alleviation of hypoperfusion, enhancement of relative cerebral blood volume, and recanalization were only observed on the stimulated side (left occipital lobe–left PCA territory). The contralateral right side, an internal control, remained ischemic due to persistent occlusion, highly suggestive of a true biological effect of stimulation. A, Acute infarctions of bilateral occipital regions (diffusion-weighted image [DWI] hyperintensities); hypoperfusion of bilateral PCA territories (time-to-maximum [Tmax] >6 seconds); symmetric bilateral relative cerebral blood volume (rCBV); bilateral PCA occlusions on MRA. B, A selection of PCA HD montage with current models predicting the e-field concentration over the ischemic region. C and D, Evolving bilateral occipital lobes acute infarcts (DWI hyperintensities); early and sustained alleviation of left occipital hypoperfusion; early and sustained rCBV enhancement of left occipital region; early and sustained left PCA partial recanalization. MRA indicates magnetic resonance angiography; MRI, magnetic resonance imaging.

**Figure 3.**
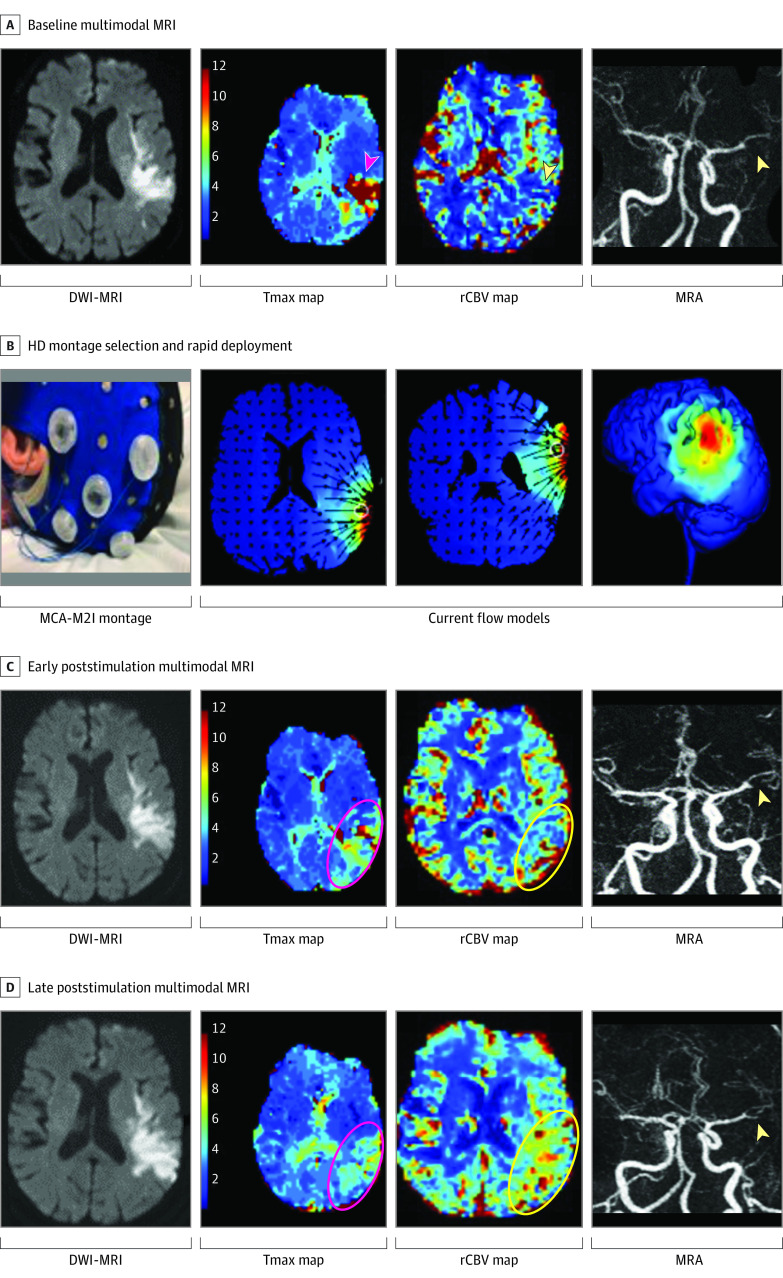
Second Exemplar of a Transcranial Electrical Stimulation in Stroke Early After Onset Clinical Trial Patient Patient 2 was a female in her 70s with acute ischemic stroke (AIS) due to left middle cerebral artery (MCA) inferior division occlusion. She received 20 minutes of 1 milliamp (mA) high-definition (HD) cathodal transcranial direct current stimulation to the left parieto-temporal region. Early alleviation of hypoperfusion region, enhancement of relative cerebral blood volume, and recanalization were observed on the stimulated side. A, Acute infarction of the left parieto-temporal region (diffusion-weighted image [DWI] hyperintensity); hypoperfusion of the parieto-temporal region (time-to-maximum [Tmax] >6 seconds); normal to low left parieto-temporal relative cerebral blood volume (rCBV); left MCA-M2 occlusion. B, A selection of MCA-inferior division HD montage with current model predicting the e-field concentration over the ischemic region. C and D, Evolving left parieto-temporal acute infarct (DWI hyperintensities); early and sustained alleviation of hypoperfusion; early and sustained rCBV enhancement of the stimulated site; early and lasting left MCA-M2 partial recanalization. MRI indicates magnetic resonance imaging.

The flow of patients screening, enrollment, and follow-up is shown in Figure 1. A total of 10 patients, 7 active and 3 sham, were enrolled in the UCLA Emergency Department, Neuro-Intensive Care and Stroke Units, from October 2018 to July 2021 (eTable 2 in Supplement 2). The first 4 patients (3 active, 1 sham) were enrolled at dose tier 1 and received 1 milliamp (mA) of HD C-tDCS for 20 minutes; the subsequent 6 patients (4 active, 2 sham) were enrolled at tier 2 and received 2 mA of HD C-tDCS for 20 minutes. Patient entry characteristics are in eTable 3 in Supplement 2. In active and sham patients mean (SD) age was 75 (20) years and 77 (12) years, the proportion of female patients was 7 of 10 (70%) and 1 of 3 (33%), and mean (SD) entry NIHSS was 8 (8.5) and 7 (2.6) (eTable 3 in Supplement 2). Scans of exemplary patients are shown in Figure 2 and Figure 3.

The speed of HD C-tDCS implementation was a median (IQR) 12.5 minutes (9-15 minutes) in the last 4 enrolled patients. The primary tolerability end point was met with all patients completing the assigned stimulation period. For the secondary tolerability end point, no discoloration or rash was detected on skin visual inspection after the stimulation. Only 1 tier 1 patient complained of mild skin burning, which was alleviated after a short pause of the stimulation.

One SICH occurred in 1 active patient at tier 2 of the study who was later determined to be a protocol deviation for not having fully met the entry criteria (stroke due to septic embolization). Nonetheless, tier 2 of the study was extended to enroll more patients. As recruitment was slower than expected, related to the COVID-19 pandemic, the study was stopped after enrollment of the 10th patient (eTable 4 in Supplement 2).

In the per-protocol exploratory efficacy analysis, imaging biomarkers of neuroprotection and collateral enhancement were characterized in 5 active patients and 3 sham patients, early and late poststimulation. The exploratory per protocol analysis of imaging end points excluded 2 active group patients with protocol deviations: 1 patient with no penumbra present at baseline on imaging core review and 1 with septic embolization as stroke cause.

**Figure 4.**
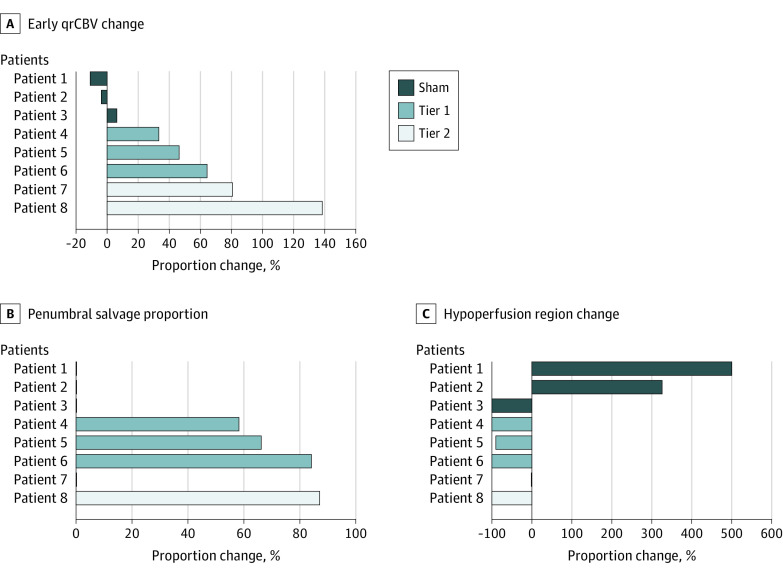
Imaging Biomarkers of Neuroprotection and Collateral Enhancement Comparing Dosage Tiers A, The enhancement of quantitative cerebral blood volume (qrCBV) in active patients early poststimulation with a dose-response pattern; B, the overall greater penumbral salvage in active vs sham patients; C, the overall greater hypoperfusion lesion (time-to-maximum >6 seconds) reduction in active vs sham patients.

The hypoperfused region was reduced by a median (IQR) 100% (46% to 100%) in the active group vs increased by 325% (112% to 412%) in sham. Change in qrCBV early poststimulation was a median (IQR) 64% (40% to 110%) in active vs −4% (−7% to 1%) in sham patients. Penumbral salvage in the active C-tDCS group was a median (IQR) 66% (29% to 80.5%) compared with 0% (IQR 0% to 0%) in sham. For the imaging biomarker of collateral enhancement (qrCBV), the response was consistent with a dose-response effect, highest at tier 2, intermediate at tier 1, and lowest at sham (Figure 4). The 24-hour vessel recanalization rate was 80% in active vs 33% in sham (eTable 5 in Supplement 2).

In a per-protocol exploratory clinical efficacy analysis, in the active group 3 patients had mRS between 0 and 2 and 2 had mRS of 3 at 90 days. Among the 3 sham patients, 2 had mRS between 0 and 2 and 1 an mRS of 6 at 90 days (eTable 6 in Supplement 2).

## Discussion

The present study is, to our knowledge, the first-in-human assessment of targeted HD C-tDCS as a potential emergent therapy for AIS. Electrical current delivery used HD individualized montages predesigned to cover distinct ischemic territories due to occlusion of 6 major arteries in the anterior and posterior intracranial circulation. We found that HD C-tDCS can be efficiently deployed in hyperacute emergency and intensive care settings.

We observed higher rates of penumbral tissue salvage and alleviation of hypoperfused ischemic regions in active patients compared with sham. These findings were accompanied by marked enhancement of rCBV and a higher rate of early recanalization poststimulation in active patients. In 1 active patient with bilateral ischemia of occipital lobes due to bilateral posterior cerebral artery (PCA) occlusions, penumbral salvage, enhancement of rCBV, and early recanalization were only observed on the stimulated side while the contralateral side, serving as an internal control, remained hypoperfused and the vessel remained occluded highly suggestive of a genuine biological effect of stimulation (Figure 2 and Figure 3). Two possible contributing mechanisms to our findings are: (1) direct anti-excitatory, anti-apoptotic, and anti-inflammatory effect of C-tDCS leading to cytoprotection of ischemic tissues; (2) electrical field–induced collateral enhancement providing retrograde reperfusion and enhanced recanalization with orthograde reperfusion of ischemic fields. The dose-related rCBV augmentation early following stimulation provides direct support for collateral enhancement as a mechanism through which HD C-tDCS exerts its alleviating effect in acute ischemia (Figure 4).^9,10,11,12^ Collateral enhancement may have contributed to higher vessel recanalization by increasing delivery of endogenous thrombolytic to both the proximal and distal ends of the clot.^13,14,15,16^ Electrical current delivered transcranially has the highest concentration in the subarachnoid space where the leptomeningeal (also known as pial) collateral vessels reside.^17,18^ In pilot testing of non–HD C-tDCS, patients with cortical strokes, who are most likely to benefit from leptomeningeal collateral pathways, showed the strongest signal of potential benefit.^19^ The collateral enhancing effect of electrical current is attributed to the activation of peri-vascular neurons and endothelial lining rich with predominantly vasodilatory neuropeptides and ion channels such as nitric oxide (NO), calcitonin gene-related peptide, and ATP-sensitive potassium.^6^

### Limitations

This study has several limitations. First, because of slow enrollment related to COVID-19 pandemic, the study was stopped early with sufficient power to address feasibility and tolerability aims, but only partially explore the safety aim. Second, this was a single-center study. A subsequent multicenter trial is planned. Third, the administering technician was not masked to the study assignment, but use of sham control enabled blinding of patients, treating team, and final outcome assessor.

## Conclusion

HD C-tDCS is a noninvasive targeted acute ischemic stroke treatment strategy that can be efficiently applied in emergency settings. Our results suggest beneficial effects upon penumbral salvage, hypoperfusion region, and qrCBV. The enhancement of qrCBV poststimulation followed a dose-response pattern suggesting collateral enhancement as a mechanism through which HD C-tDCS may alleviate ischemia and possibly promote vessel recanalization. Our data warrants conduct of larger safety and efficacy trials of HD C-tDCS as an AIS therapy and acute stroke-specific refinements in HD equipment design to further accelerate deployment time.

## Notes

Supplement 1.
Study Protocol

Supplement 2.
eMethods.
**eTable 1.** Entry Criteria **eTable 2.** Reasons for Patients' Ineligibility for Intravenous Thrombolysis and Endovascular Thrombectomy **eTable 3.** Demographic and Clinical Patient Features at Baseline **eTable 4.** Main Outcomes Analysis **eTable 5.** Per Protocol Exploratory Imaging Analysis **eTable 6.** Per Protocol Exploratory Clinical Analysis

Supplement 3.
Nonauthor Collaborators

Supplement 4.
Data Sharing Statement
