# Supplementary material for: High-definition Cathodal Direct Current Stimulation for Treatment of Acute Ischemic Stroke: A Randomized Clinical Trial
Source: JAMA Netw Open. 2023 Jun 21;6(6):e2319231. doi: 10.1001/jamanetworkopen.2023.19231 (PMC10285579; doi:10.1001/jamanetworkopen.2023.19231)
Supplement: Supplement 1. — Study Protocol [file jamanetwopen-e2319231-s001.pdf]

Study Protocol

Transcranial electrical Stimulation in Stroke EaRly After onset Clinical Trial (TESSERACT)

Version 1.6 (Revised)
1/12/21

Investigational Plan:
Protocol: Transcranial electrical Stimulation in Stroke EaRly After onset Clinical Trial (TESSERACT)

Overview of Protocol of TESSERACT

Title	Transcranial electrical Stimulation in Stroke EaRly After onset Clinical Trial (TESSERACT)
Objective	To evaluate the safety, tolerability, and feasibility, and preliminarily explore the potential efficacy, of transcranial direct current stimulation (tDCS) as an innovative non-invasive neuroprotection technique in acute ischemic stroke.
Study Design	A single site, sham- controlled, dose escalation study
Population Studied	24-48 acute ischemic stroke patients from Ronal Regan Medical Center (RRMC) Emergency Department or RRMC Inpatient hospital with:<br>1) New focal neurologic deficit consistent with acute ischemic stroke; 2) NIHSS >4; 3) Age >18; 4) Last known well time within 24 hours of randomization stimulation initiation; 5) ICA or M1 or M2 MCA occlusion on MRA; 6) substantial salvageable penumbra on multimodal MRI (tissue-at-risk perfusion lesion volume exceeds core volume by ≥ 20%); 7) Ineligible for intravenous thrombolysis; 8) Ineligible for endovascular thrombectomy; 9) Signed informed consent obtained from the patient or patient's legally authorized representative.
Intervention	3:1 randomization to active treatment vs sham control
Device	Transcranial Direct Current Stimulation (tDCS)

A. Specific aims

Transcranial direct current stimulation (tDCS) is a non-invasive neuromodulatory technique that applies a weak electrical current to the brain via scalp electrodes. tDCS has been investigated in a variety of chronic neurological and neuropsychiatric disorders such as depression, traumatic brain injury and stroke rehabilitation with promising benefits and excellent safety profile. Notably, tDCS has been shown to carry a strong neuroprotective effect in animal models of acute ischemic stroke (AIS) due to large vessel occlusion (LVO).

Stroke is a leading cause of mortality and morbidity across the world. While endovascular therapy (ET) has fundamentally improved outcomes of AIS patients with LVO, patient outcomes even with ET remain suboptimal, with only 20-25% achieving a disability-free outcome. Complementary neuroprotective, vasoprotective, and collateral enhancement strategies can preserve cells and the neurovascular unit until orthograde reperfusion is achieved by ET, particularly in AIS patients in whom ET is delayed due to hospital-to-hospital transfer. Furthermore, these agents may protect against reperfusion injury, and attenuate secondary injury cascades that persist despite reperfusion. Importantly as well, even in the modern thrombectomy era, not all patients with LVO are treatable with ET. In some patients with LVOs, ET is not technically performable due to unfavorable angioarchitectural features or in other LVO patients, the risk of intervention is judged too great, due to the presence of early hemorrhagic transformation, temporally advanced or large core infarct, or severe cardiorespiratory comorbidities. Therefore, a great need for complementary or standalone therapies exist. While many past attempts at developing neuroprotective therapies have been disappointing, the great preponderance were pharmacologic agents that affected only one or two molecular ischemic pathways and were administered intravenously, reducing delivery to brain areas with low blood flow, and, even there, requiring passage through the blood-brain barrier. In contrast, in pre-clinical acute ischemic stroke models, tDCS has been shown to have widely pleiotropic neuroprotective molecular mechanisms of action and can be delivered to the salvageable ischemic tissue beyond the occlusive thrombus despite reduced blood flow. Given the promising results in acute stroke models, translational studies to the acute human ischemic stroke patients are needed, beginning with a dose-escalation feasibility and safety trial.

In this proposal, we will perform a single center, sham-controlled, dose escalation study. Cathodal tDCS (C-tDCS) will be delivered to penumbral tissue in patients with large and medium vessel occlusions who are not eligible for endovascular thrombectomy, yet harbor substantial salvageable penumbra.

Specific Aim I: To establish the safety and tolerability of tDCS in AIS.

Hypothesis I-a: tDCS will be safe in AIS patients with no increased risk of symptomatic intracranial hemorrhage (SICH) within the 24-hour period after stimulation. Hypothesis I-b: tDCS will not increase the rates of asymptomatic intracranial hemorrhage, early neurological deterioration (NIHSS during the 24-hour period after stimulation), 90-day mortality, and all serious adverse events. Hypothesis I-c: Majority of AIS patients (≥ 90%) will tolerate the tDCS by completing at least 75% of the stimulation period with no major stimulation related pain, discomfort and skin changes.

Specific Aim II: To determine the feasibility of delivering tDCS in AIS.

Hypothesis II-a: tDCS will be started more rapidly over the course of the study and in the last 10 patients median time from randomization to tDCS initiation will be ≤ 10 minutes.

Specific Aim III: To explore the potential imaging and clinical efficacy of tDCS in AIS.

This aim will be accomplished by measuring, in actively treated and sham patients:

1) Early and late penumbral salvage, defined as proportion of penumbral tissue on baseline multimodal MRI that does not proceed to infarct on 2-hour and 24-hour MRI (PWI); **2)** Early and

late collateral enhancement, evidenced by reduced perfusion lesion volume on 2-hour and 24-hour perfusion-weighted MRI (PWI); **3)** Early and late reduction in infarct growth from baseline to 2-hour and 24-hour MRI; 4) Early improvement in neurologic deficit (NIHSS at 24h); **5)** Improvement in final 3-month disability and quality of life (modified Rankin scale, AMC Linear Disability Scale, EQ-5D, and Barthel Index)

Impact

This in-hospital safety trial, in non-thrombectomy candidates in a closely-observed, Emergency Department and hospital settings, will identify tDCS dose regimens that are feasible, well-tolerated, and preliminarily safe in patients with acute ischemic stroke. Study finding will identify the most promising dose regimens to advance to phase 2b and pivotal phase 3 efficacy trials for two patient populations with pressing need for improved therapies: 1) thrombectomy ineligible patients (standalone neuroprotection), and 2) thrombectomy-eligible patients (bridging neuroprotection in prehospital and early ED-arriving patients).

B. Background and Significance

Stroke is a devastating condition and continues to be a leading cause of adult death and disability.[1] Current therapeutic strategies for AIS focus on timely restoration of blood flow by recanalization of the occluded artery to salvage penumbral tissue, using pharmacologic fibrinolysis and/or endovascular thrombectomy (ET).[2,3] However, the reperfusion strategy has limitations that would be aided by a neuroprotective intervention. An important minority of early-arriving patients are ineligible for both IV tPA (due to lytic contraindications) and ET (due to difficult vascular access, large core, peripheral target occlusion beyond catheter reach in patients with medium vessel occlusion (MVO)). Other patients are non-responders: IV tPA only achieves reperfusion in only 15-40 percent of large vessel occlusions (LVOs); ET fails to achieve substantial reperfusion in 20-30%. Furthermore, even among reperfusing patients, rates of excellent outcome are low, only 20-25% mRS 0-1, due to infarct growth prior to, and following reperfusion. [4-6] Therefore, even in the modern stent retriever era, the need to develop additional therapies exists. Neuroprotective therapies interrupt the cellular, biochemical and metabolic process that mediate hypoxic and reperfusion cellular injury. Neuroprotective therapies could serve as alternative treatments for patients who harbor salvageable penumbra in whom reperfusion therapies are contra-indicated or have failed, and as early bridging therapy in pre-reperfusion patients, preserving more viable penumbra to rescue intervention by reperfusion. While multiple candidate neuroprotective agents have failed in translation from animal to human studies, important lessons have been learned.[7,8] Two intervention properties that offer greater prospect of success are: 1) pleiotropic effects, interdicting multiple pathways in the ischemic cascade, and 2) delivery by a direct transcranial, rather than intravascular, route, with fast and direct delivery to neural tissues regardless of cerebral perfusion. Transcranial direct current stimulation (tDCS) offers promise as just such an approach. Furthermore, few trials of neuroprotection in human have used penumbral imaging for patient selection [9] and importantly, the recent trials of thrombectomy in late-arriving patients have shown a significant benefit from recanalization therapy in patients with a salvageable penumbra despite their late presentation from last known well time. [10,11]Therefore, the use of penumbral imaging to limit the patient selection to those for whom the intervention is most likely to be of benefit is crucial in studies of neuroprotection and our study utilizes such an approach.

tDCS is a non-invasive neuromodulation method that delivers a weak electrical current to the brain via scalp electrodes.[12] Rather than directly eliciting a neuronal response, tDCS modulates neuronal excitability in regions of the brain depending on the polarity of stimulation. By altering the resting membrane potential cathodal tDCS (C-tDCS) reduces neuronal excitability [in contract to anodal tDCS (A-tDCS) which increases excitability]. [13,14] tDCS has been extensively investigated in humans for decades, as a neuromodulatory intervention to treat depression and diverse other neuropsychiatric disorders, as a neuroplasticity-enhancing intervention for chronic stroke patients, and as a tool to change cognition and behavior in healthy individuals. [15-18]Thus far, tDCS has been found safe and tolerable with no reported serious adverse events across multiple clinical and preclinical studies. [19,20] In addition to these established applications of tDCS, tDCS is of substantial promise for acute cerebral ischemia, based on preclinical studies. In pre-clinical studies, multiple investigators in several independent labs worldwide have found evidence of a neuroprotective effect of tDCS in different animal models of acute cerebral ischemia (table 1):

B.1. Proposed neuroprotective mechanisms of tDCS based on experimental pre-clinical models of acute cerebral ischemia

B.1.1. Inhibition of peri-infarct excitatory depolarizations

During the acute stages of cerebral ischemia, an excitotoxic cascade will be triggered by the excess glutamate and other excitotoxic amino acids that are released as the result of cellular necrosis.[21] Experimental models of acute middle cerebral artery occlusion (MCAO) have shown that the excitotoxicity generates recurrent spontaneous waves of depolarization also known as peri-infarct depolarizations (PIDs).[22,23] The PIDs occur soon after the MCA occlusion and spread across the penumbra to the normally perfused tissue. The infarct growth correlates with the number and duration of PIDs and the basis for this relationship has been related to: 1) An abnormal vasoconstriction in response to depolarization 2) An imbalance between increased metabolic overload, induced by the depolarization, and blood supply in acute ischemic stroke.[24] Notturno et al. studied the effect of C-tDCS on PIDs in 3-vessel occlusion rat stroke model, with cumulative stimulation durations of 120 and 180 mins (15' on-15' off cycles).[25] They found that C-tDCS was applied to the ischemic MCA territory significantly reduced PIDs, and reduced infarct volume by 20-30%. They found no effect on brain edema between the stimulated and sham groups and no tDCS induced macroscopic or microscopic lesion or hemorrhagic transformation.

B.1.2. Anti-inflammatory, Anti-apoptotic, and Angiogenic effect

Beyond the activation of the excitotoxic cascade, an inflammatory response and programmed cellular apoptosis will result in a secondary damage and expansion of the irreversibly damaged core (Figure1). [21] Therefore, suppression of the innate pro-inflammatory cells and the cellular apoptotic cascade results in the reduction of the infarct size and cerebral edema. Peruzzotti et al. studied tDCS in acute stroke mice models with MCAO with cathodal hemispheric tDCS applied for 40 minutes (20'on-20off-20'on). [26] Cathodal stimulation of the ischemic hemisphere reduced final infarct size, with lowering of cortical glutamate synthesis, downregulation of N-methyl-D-aspartate (NMDA) receptor (NR2B) expression, and reduction in peri-ischemic inflammatory response and apoptotic markers.

Furthermore, a significant functional amelioration and improvement of cerebral edema were observed even when stimulation was applied hours after the MCAO. No macroscopic or microscopic lesion or hemorrhagic transformation induced by tDCS was found.

Baba et al. showed in their study that a low-frequency (2 and 10 Hz) electrical cortical stimulation exerts neuroprotective effects reflecting by attenuation in both necrotic and apoptotic cell deaths, blockade of microglial/astrocytic activation and expression of trophic factors. They also demonstrated an increase in cerebral blood flow of the electrically stimulated animals. [27] No neuroprotective effect was observed at higher frequency (50Hz).

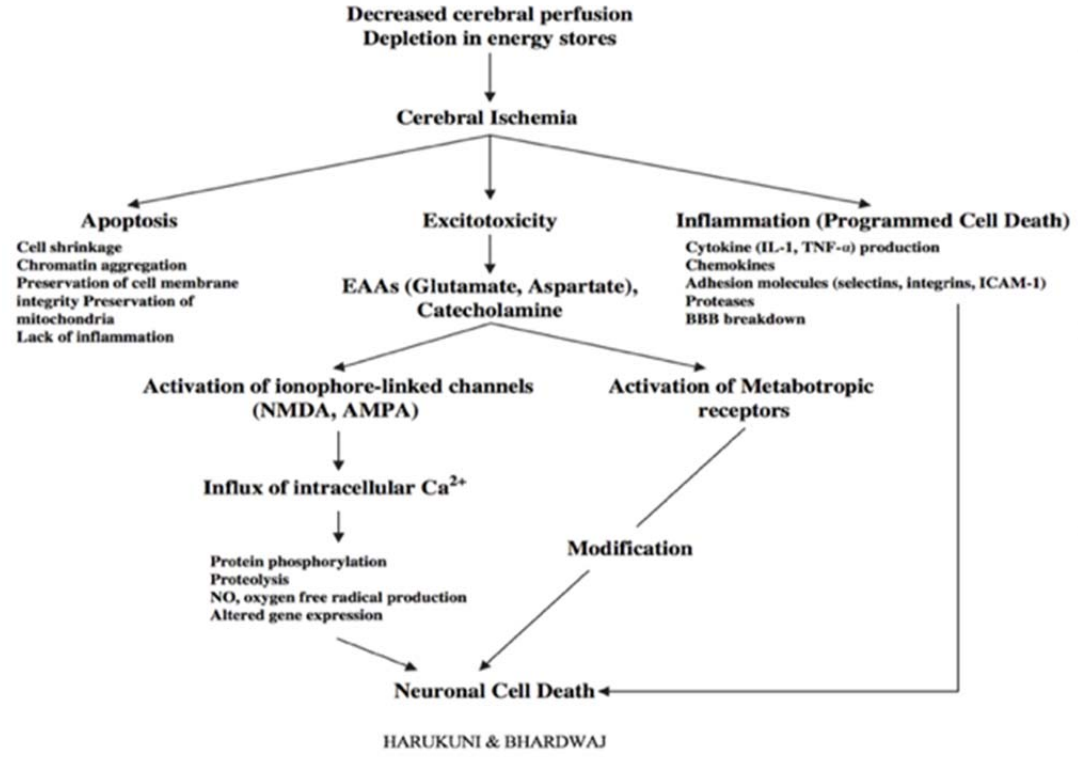

Figure 1. This schematic shows the events leading to ischemic brain injury.

B.1.4. Preservation of neuronal axons

Kim et al. studied the neuroprotective effect of cathodal and anodal tDCS on axons and myelin integrity. They delivered tDCS for 30 minutes two days after MCAO. They found significant amelioration of axonal damage and preservation of white matter axonal integrity in rat models of cerebral ischemia. They demonstrated such effect only after anodal stimulation. [28]

B.1.3. Direct vasodilatory effect

In addition to the direct neuroprotective effects, tDCS may confer benefit in AIS via direct vasodilation and collateral blood flow enhancement. In fact, Fox et al. found a direct vasodilatory effect of electrical stimulation when applied over the basilar artery. [29]This effect was more pronounced with cathodal compared to anodal stimulation.

Study authors	Type of subjects	Onset of stim following MCAO	Polarity and location of the center electrode	Intensity (mA)	Total Duration of stim	Total N
**Notturno et al et. al 2013**	Rat MCAO model	45 min	Cathode over cortical surface overlying ischemia	0.2 (density of 2.86 mA/cm^2)	120 and 180min (alternating 15'on-15'off)	48
**Peruzzotti-Jametti et. al 2010**	Mice MCAO model	30 min	Cathode or anode over cortical surface overlying ischemia	0.25 (density of 5.5 mA/cm^2)	40min (alternating 20'on-20'off)	137
**Baba et al. 2009**	Rat MCAO Model	60 min	Electrode over cortical surface overlying ischemia	0.1 and 0.2 with frequencies of 0, 2, 10, 50	3 Days or 1 week	107
**Kim et al. 2010**	Rat MCAO model	Day 2	Cathode or anode over cortical surface overlying ischemia	0.1	30 min daily for 2 weeks	61
**Glickstein et. al 1999**	Rat MCAO model	5 days prior to MCAO	Stimulation of the fastigial nucleus of cerebellum	0.01-0.02	60min(alternating 1 sec on-1 sec off)	98
**Reis et.al 1997, 1991**	Rat MCAO model	Immediately after, up to 10 days prior to MCAO	Cathode over the fastigial nucleus of cerebellum	0.01-0.02	60min	99
**Zhang et.al 1993**	Rat MCAO model	3-5 min	Cathode over the fastigial nucleus of cerebellum	0.07-0.1	60min (alternating 1 sec on-1 sec off)	44
**Yamamoto et.al 1993**	Rat MCAO model	Immediately	Cathode over the fastigial nucleus of cerebellum	0.07-0.1	60min (alternating 1 sec on-1 sec off)	41
**Burger et. al 1990**	Rat MCAO model	Immediately	Stimulation of the fastigial nucleus of cerebellum	Unknown	60min	

Table 1. shows the detailed description of the studies using tDCS as a neuroprotective method.

C. Preliminary studies

C.1. Experience with transcranial neuromodulation in human subjects

Our group at UCLA has extensive experience with transcranial neuromodulation using direct electrical and magnetically induced currents in human subjects. Over the past 10 years, we have performed transcranial neuromodulation in over 500 subjects.[30-35] This substantial experience provides a firm foundation for undertaking tDCS studies in patients with acute ischemic stroke.

C.2. A meta-analysis of preclinical studies using tDCS as neuroprotection in acute cerebral ischemia

To assess the neuroprotective effect of tDCS in AIS, we performed a systematic review of all preclinical acute cerebral ischemia studies using tDCS as a neuroprotective method. Our systematic search identified 21 controlled comparisons of tDCS in preclinical acute cerebral ischemia models, including a total of 256 animals, all with middle cerebral artery occlusion. [25,26,36-40] Hemispheric cathodal stimulation was used in 3 experiments (32 animals), hemispheric anodal stimulation in 1 experiment (8 animals), Electrical stimulation in 4 experiments (--- animals) and fastigial nucleus stimulation in 13 experiments (91 animals). Overall, tDCS reduced final infarct volume by 24.68 mm^3 (95% CI 26.53-22.83, $P<0.00001$). Only mild heterogeneity of effect by stimulation type was noted (I^2=90.5%), with the infarct reduction magnitudes relatively larger with

cathodal hemispheric stimulation– 30.94 mm ^{3} (95% CI 34.35- 27.53, P< 0.00001), followed by targeted fastigial nucleus stimulation – 26.4 mm ^{3} (95% CI 29.26-23.63, P< 0.00001), then hemispheric electrical stimulation – 18.16 (95% CI 23.85-12.48, P< 0.00001), and hemispheric anodal stimulation- 13 mm ^{3} (95% CI 17.56- 8.44, P< 0.00001) (Figure 2).

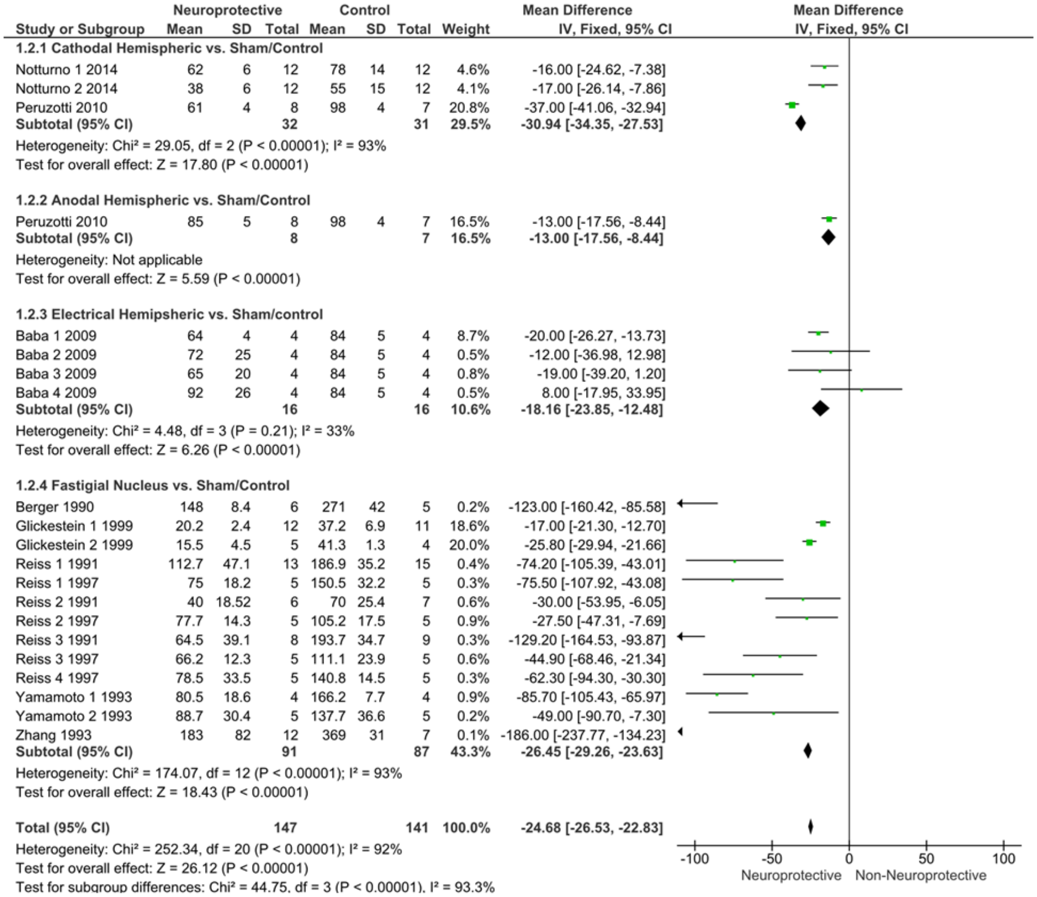

Figure 2. Forest plot shows the neuroprotective effect of tDCS across 21 pre-clinical experiments*
*includes all published studies except Kim et al 2009, which did not report infarct volume in assimilable units

These findings demonstrate that tDCS significantly reduces final infarct volume across animal preclinical studies. The greatest neuroprotective effect is observed with the C-tDCS directly applied to the ischemic hemisphere (hemispheric cathodal stimulation). Therefore, our proposed study of hemispheric C-tDCS in patients with acute ischemic stroke is warranted.

C.3. Vascular response associated with tDCS

A preliminary study in human volunteers by Marom Bikson (Co-mentor of the current proposal) demonstrated that tDCS produces vasodilation of cerebral vessels (Figure 3). Furthermore, the vasodilatory response to tDCS is evident by its known mild dose-dependent effect in causing skin erythema. The cerebral vasodilatation is likely partially due to non-specific polarization of vascular system. Bikson and colleagues also demonstrated that 10 minutes of tDCS resulted in up-regulation of endothelial nitric oxide synthase (eNOS) gene expression and increase production of nitric oxide (NO), a

known vasodilator. [41] Samdani et al. have demonstrated in their study that the endothelial NO upregulation favorably affects outcomes by the accentuation of the cerebral blood flow and attenuation of platelet aggregation, platelet adhesion, and NMDA current. [42] These findings support that tDCS could augment blood flow through the stimulated vasculature. Leptomeningeal collateral networks, peripherally located, are particularly accessible to the electric field. The potential vasoactive effects of tDCS also raise the possibility that stimulation will affect the blood-brain barrier. While no hemorrhagic transformation with C-tDCS was noted in preclinical models, this potential effect supports the approach of undertaking dose escalation safety trial as the first study in AIS patients, even though all studied dose tiers are within ranges safe in chronic stroke and other brain disease patients.[12 20]

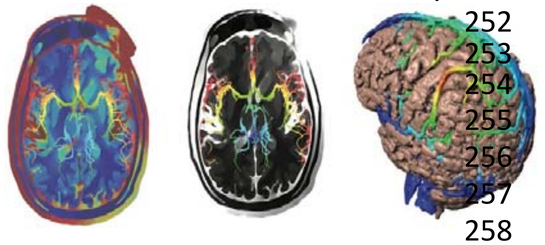

Figure 3. shows models of intra-cranial current flow during tDCS is concentrated by the vasculature

C.4. Feasibility of neuroprotection therapies in acute ischemic stroke

Our group has extensive experience with the conduct of clinical trials of drug and device neuroprotective therapies for acute ischemic stroke, both as standalone interventions and as a complement to reperfusion treatment. We recently completed the NIH Field Administration of Stroke Therapy-Magnesium phase 3 trial, enrolling 1700 patients. [8] Of particular relevance to the current proposal, we have conducted trials of transcranial delivery of acute neuroprotective intervention using laser [43], and trials of collateral enhancement interventions [44] and trials and studies using MRI penumbral imaging and permeability imaging as technical efficacy and safety end points.

C.5. Frequency and features of AIS LVO and MVO patients with substantial salvageable penumbra who do not undergo ET

Among the eventual target populations for tDCS as a neuroprotective intervention in acute LVO and MVO AIS, several are suboptimal for inclusion in an initial, dose escalation safety trial. Ambulance patients (prehospital neuroprotection) are in a chaotic environment unsuited for close safety observations; ED patients pre-ET (bridging neuroprotection) have intense time-pressure for usual care making safety-emphasis studies difficult; ET failure patients (rescue neuroprotection) have potential complications from their failed procedure confounding study interpretation. In contrast, ET-ineligible patients (pure neuroprotection) are an attractive cohort for a safety study, as they are under close observation, free of intense time-pressure for ET, and free of course outcome being strongly determined by ET outcome. To plan the current proposal, we, therefore,

performed a study to delineate the frequency, clinical characteristics, and outcomes of patients with substantial salvageable penumbra who are ineligible for ET in the modern stent retriever era [45]. Patients were recognized as having substantial salvageable penumbra when their perfusion lesion volume (tissue with a delay in contrast arrival to peak concentration (Tmax) of ≥ 6 sec on perfusion-weighted image (PWI)) was ≥ 1.2 times larger than the ischemic core volume (tissue with a low mean water diffusivity or apparent-diffusion coefficient (ADC) ≤ 620 μm^2/s on diffusion-weighted image (DWI))

(PWI-DWI Mismatch). [46-48] We then categorized the patients with substantial salvageable penumbra as having an LVO if MRA or CTA showed occlusion of the intracranial internal carotid (ICA), the M1 segment of the middle cerebral artery (MCA), the vertebral artery (VA), or the basilar artery (BA). Patients were categorized as having an MVO if MRA or CTA showed occlusion of the M2 segment of the MCA, the A1 segment of the anterior cerebral artery (ACA), or the P1 segment of the posterior cerebral artery (PCA); or if perfusion imaging indicated occlusion an M3 segment of the middle cerebral artery by showing a perfusion lesion volume of at least 10 ml in an appropriate territorial distribution. Among 174 consecutive AIS patients, 29 (17%) were LVO and MVO patients with substantial salvageable penumbra who did not undergo ET. Mean age was 81 (±13), 45 % were female, and median NIHSS was 11 (IQR 5-19). The prevalence of LVO was 59 % (19/29) and MVO 41% (12/29). Patients with ICA, M1 and M2 occlusions constituted most of the cases (78%). The four most common reasons for not pursuing ET intervention were: distal occlusion (28%), large infarct core (16%), low NIHSS (16%), temporally advanced core injury evident from fluid attenuated recovery (FLAIR) changes on MRI or frank hypodensity on CT (13%). Other reasons included: chronic occlusion of the cervical internal carotid artery precluding intracranial access (9%); poor pre-stroke baseline function (9%); intracranial occlusion judged to be a chronic atherosclerotic occlusion (6%); extracranial vessel tortuosity precluding intracranial access (3%). Median time from LKN to imaging was 410 min (IQR 198-615). Mismatch ratio was median 5.6 (IQR 2- infinite), salvageable penumbra volume mean was 54 ml (±63), and ischemic core volume was mean 20ml (±31). Severe disability or death at discharge (mRS 4-6) occurred in 72%. These findings demonstrate that, even in the modern stent retriever era, one in six acute cerebral ischemia patients presents with substantial salvageable penumbra judged not appropriate for ET. This population is more than sufficiently common for the proposed study. Figure 4 demonstrates an exemplary patient with an LVO and substantial penumbra in whom endovascular thrombectomy was not pursued.

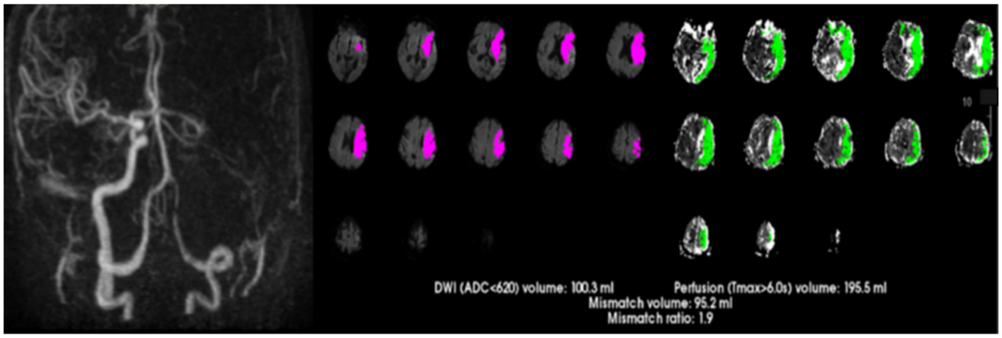

Figure 4. 92 y/o woman with left intracranial internal carotid occlusion with ischemic core judged too large for favorable benefit-risk ratio. Perfusion lesion volume 195 ml, ischemic core volume 100.3 ml, mismatch ratio 1.9. The penumbral volume is calculated as 95 ml (195 ml-100ml).

D. Trial Design and Methods

D.1. Study overview

This proposal is a prospective, single-center, dose-escalation safety, tolerability and feasibility study of tDCS in acute stroke patients with substantial salvageable penumbra

due to a large or medium vessel occlusion who are ineligible for endovascular therapy. The primary safety endpoints will be symptomatic intracranial hemorrhage during the 24-hour period after stimulation. Secondary measures of safety will include asymptomatic intracranial hemorrhage, early neurological deterioration, 3-month mortality and all serious adverse events. Tolerability will be judged based on the percentage of the patients completing the protocol-assigned stimulation treatment and secondarily, the rate and severity of cutaneous, neurologic, nociceptive or other adverse effects will be assessed. Feasibility endpoints will analyze the speed with which tDCS will be implemented. Finally, we will explore signals of potential efficacy by examining the imaging biomarkers, including penumbral salvage, collateral enhancement, and infarct growth, and clinical outcomes of early neurologic deficit evolution, and 3-month global disability and health-related quality of life.

D2. Technology

D.2.1. tDCS Device and Stimulation Parameters

The study will employ a Soterix™ high-definition DC-Stimulator, owned by the principal investigator. This tDCS unit consists of a stimulator, 4x1 HD interface and an adjustable cap with pre-maid openings that quickly and easily fits different head sizes. (Figure 5). The cap will be loaded with plastic electrode holders. These will be filled with conductance gel (Signa® gel), 1 cm^2 electrodes will be placed in the holders, and the holder then locked.

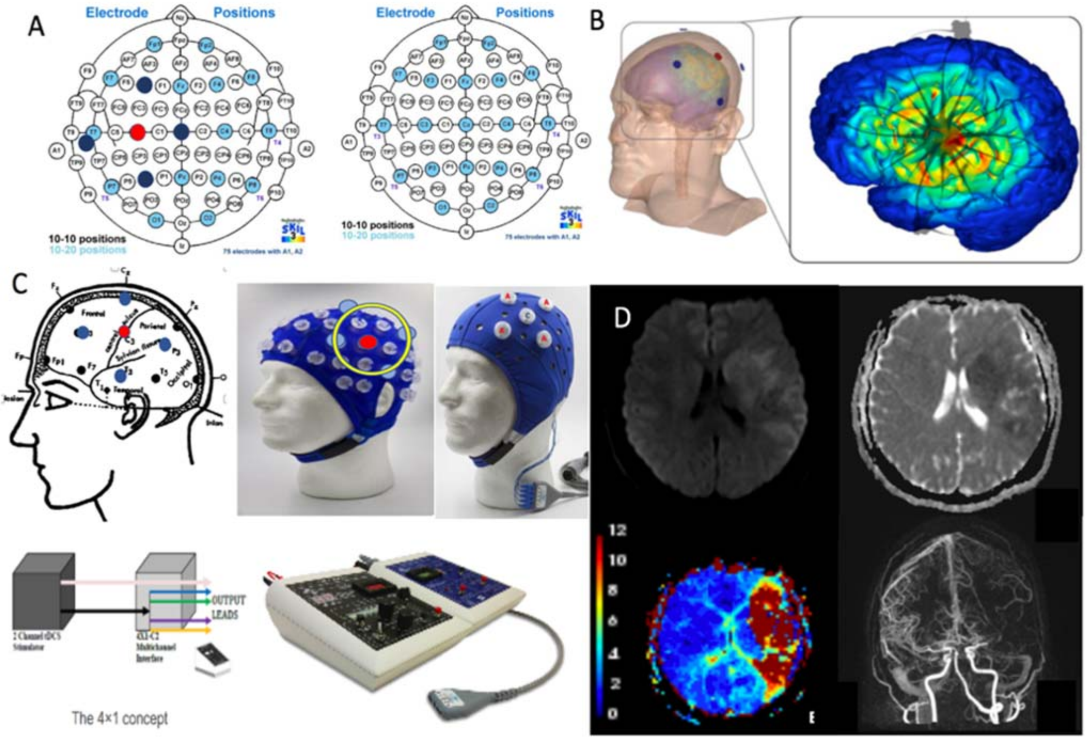

Figure 5. Shows an example of electrode positioning in a 4 to 1-ring configuration in a patient with left M1 occlusion. A) The reference electrodes (anode-blue) are positioned on F3, T3, Cz and P3 and the center electrode (cathode-red) is positioned over the C3 (central sulcus). B) shows the computational modeling of the electrical field, concentrated over the MCA territory. C) Shows the electrode positioning on the tDCS HD cap, the schematic of the tDCS stimulator connected to the HD interface, and the Soterix™ HD tDCS unit (stimulator + interface) that will be used in this study. This stimulator connects to

the adjustable cap. D) Shows the penumbral region on the perfusion MRI of the patient with L MCA occlusion.

D.2.2. Montage

The electrode positioning montage will be a 4 to 1-ring configuration, with the center or active electrode connected to cathode and the 4 reference or return electrodes connected to anode. The 4 to 1-ring configuration is chosen to focus the stimulation to the penumbral region. (Figure 6)

For electrodes positioning location, we use one of the 6 predefined positionings according to the location of the vascular occlusion: middle cerebral artery (MCA) M1 branch, MCA inferior branch (M2-I), MCA superior branch (M2-S), Anterior Cerebral artery (ACA), Posterior Cerebral Artery (PCA), Posterior Inferior Cerebellar Artery (PICA). These electrode positionings are based on computational modeling of 6 electrical fields concentrated over different parts of the aforementioned vascular territories: electrical field covering territory of MCA-M1 branch (Fig 6A, a); electrical field covering territory of MCA-M2 superior branch (Fig 6B,b); and electrical field covering territory of MCA-M2 inferior branch (Fig 6C,c), electrical field covering territory of ACA-(Fig 6D, d); electrical field covering territory of PCA (Fig 6E, e); electrical field covering territory of PICA (Fig 6F, f);

Figure 6.

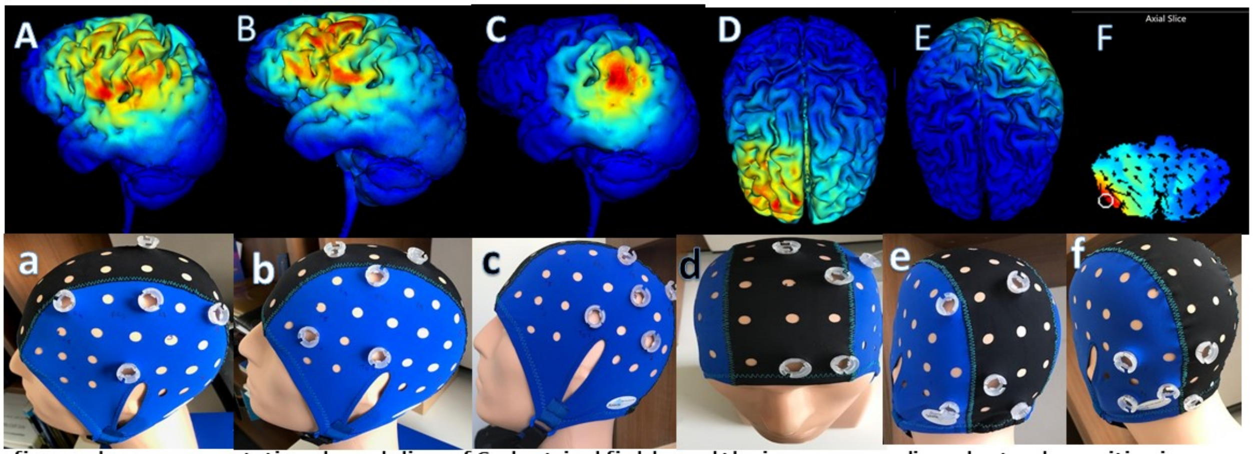

This figure shows computational modeling of 6 electrical fields and their corresponding electrode positioning covering different parts of the left Middle Cerebral Artery (MCA), Posterior Cerebral Artery (PCA), Anterior Cerebral Artery (ACA), Posterior Inferior Cerebellar Artery (PICA) territories according to the branch occlusion:
A) Electrical field covering the MCA-M1 branch territory; a) Anodes over F3, Cz, P3, T7; Cathode over C3.
B) Coverage of the MCA-M2 superior (S) branch territory; b) Anodes over C1, F1, F7, T7; Cathode over FC3.
C) Coverage of the MCA-M2 inferior (I) branch territory; c) Anode over C3, P3, T7, P7; Cathode over CP5.
D) Coverage of the ACA branch territory; d) Anodes over FPz, FCz , FC3, FP1; Cathode over F1
E) Coverage of the PCA inferior branch territory; e) Anodes over Pz, Iz, PO9, P3; Cathode over O1
F) Coverage of the PICA branch territory; f) Anodes over O1, O9, Ex1, Ex5; Cathode over PO9

D.2.3. Sterility and Quality Assurance

Prior to stimulation, all the electrodes will be monitored for any sign of damage such as chipping. The electrodes will be discarded after being exposed to 5 cycles of stimulation. We will avoid any metal to contact the electrodes. The stimulation cap and the electrodes will be sanitized prior to each subject use.

D.2.4. Device training

Device use will be performed by physician investigators, technologists, and research staff who have completed training and certification in safe tDCS use. Training will include instructions on different components of the device, installing and assembling the components, charging the device prior to the first use, recharging the device after each procedure, verifying the device is charged prior to each procedure, recognizing and addressing the different Warning/Error indications (e.g., battery light flashing blue, indicating that the battery is low but can still perform at least one procedure, battery light steady red, indicating battery low error – not enough battery power to run a complete procedure, etc.) and specific instructions for returning the device to the sponsor in the event of an error notification that cannot be addressed by the site personnel, or a failure of the device to charge after three hours. All device training will be documented in a training log that will be maintained in the site regulatory binder.

D3. Subjects

Twenty-four to 48 acute ischemic stroke patients with substantial salvageable penumbra due to a large or medium vessel occlusion who are ineligible for endovascular therapy and meet study inclusion/exclusion criteria will be enrolled from Ronald Reagan Medical Center (RRMC) Emergency Department or RRMC inpatients.

Based upon acute stroke patient referral rates to UCLA over the past 3 years and our retrospective study looking at the thrombectomy-ineligible patients with substantial penumbra in an 8-month period (1 in 6 acute ischemic stroke patients), we anticipate *8-16 enrollments per year.* Therefore, we estimate that the study enrollment will take 3 years to complete. Enrolled subjects will be randomized to active versus sham stimulation in 3:1 ratio.

D.3.1 Entry Criteria

Inclusion criteria:

1) New focal neurologic deficit consistent with AIS

2) NIHSS≥4 or NIHSS< 4 in the presence of disabling deficit (a deficit that, if unchanged, would prevent the patient from performing basic activities of daily living such as bathing, ambulating, toileting, hygiene, and eating or returning to work);

4) Age>18;

5) Presence of any cortical vessel occlusion including ICA, branches of MCA, Anterior Cerebral artery (ACA), Posterior Cerebral artery (PCA), Posterior-Inferior cerebellar artery (PICA);

6) Presence of salvageable penumbra with Tmax> 6 sec/ ischemic core volume (ADC < 620 $\mu m^2/s$ or rCBF< 30%) ≥ 1.2

7) Patient ineligible for IV tPA, per national AHA/ASA Guidelines

8) Patient ineligible for ET per AHA/ASA national Guidelines – one or more of: poor prestroke functional status (mRS score >1), mild neurological symptoms (NIHSS <6), large ischemic core (ASPECTS <6), thrombectomy not technically performable due to severe vessel tortuosity, cervical artery chronic occlusion, or other unfavorable angioarchitectural features that preclude endovascular access to the target intracranial vessel.

9) Subject is able to be treated with tDCS within 24 hours of last known well time;

10) A signed informed consent is obtained from the patient or patient's legally authorized representative

Exclusion criteria

1) Acute intracranial hemorrhage

2) Evidence of a large Ischemic core volume (ADC < 620 μm^2/s or rCBF< 30%) ≥ 100

3) Presence of tDCS contraindications - electrically or magnetically activated intracranial metal and non-metal implants.

4) Severe MR contrast allergy or renal dysfunction with eGFR<30ml/min, precluding MRI gadolinium or CT iodine contrast

5) Pregnancy

6) Signs or symptoms of acute myocardial infarction, including EKG findings, on admission

7) History of seizure disorder or new seizures with presentation of current stroke

8) Evidence of any other major life-threatening or serious medical condition that would prevent completion of the study protocol including attendance at the 3-month follow-up visit

9) Concomitant experimental therapy

10) Preexisting scalp lesion at the site of the stimulation or presence of skull defects (may alter current flow pattern)

11) Preexisting coagulopathy, consist of platelet count of ≤ 100, INR ≥ 3, PTT ≥ 90.

12) Patients suspected of having infective endocarditis and ischemic stroke related to septic emboli

13) Patients suspected or known to be infected with coronavirus 2019 (CoVID-19)

14) Patient with radiographic evidence or suspicion of chronic conditions that may predispose them to intracranial hemorrhage including brain arteriovenous malformations, cerebral cavernous malformations, cerebral telangiectasia, multiple previous intracerebral hemorrhages (amyloid angiopathy)

15) Suspected cerebral vasculitis based on medical history and CTA/Magnetic Resonance Angiogram (MRA)

16) Suspected cysticercosis

17) Suspected cranial dural arteriovenous fistula

18) Cerebral venous thrombosis

19) Head trauma causing loss of consciousness, concussion, confusion, or a headache within the past 30 days

20) Patient has suffered a hemorrhagic or ischemic stroke within the last three (3) months

21) History of a cancer known to cause hemorrhagic metastases, e.g., melanoma, renal cell carcinoma, choriocarcinoma, thyroid carcinoma, lung carcinoma, breast carcinoma, and hepatocellular carcinoma

22) History of left atrial myxoma

23) Evidence of dissection in the intracranial cerebral arteries

24) Suspicion of aortic dissection

25) Significant mass effect with midline shift

26) The patient is in a coma

Biological variables

The entry criteria have been designed to be broadly inclusive of biological variables that may modify disease course and treatment response, including enrollment of all adults of any age, both males and females (except pregnant females), and all weights compatible with MR-scanning. Pregnant women will be excluded as the safety of tDCS

has not been established in pregnancy. A pregnancy test will be performed prior to enrollment in women of childbearing age. Children will be excluded because of the rarity of diagnosis in children in the acute time window, and greater brain plasticity and recovery in younger individuals. Given the uncommon availability for enrollment and very different course, including children in trial could differentially favor or unfavor one study arm, and make interpretation of findings challenging.

With regards to the time window, patients treatable within 24 hours of last known well are included, as the DAWN and DEFUSE 3 trials have shown that patients in the 6-24h window with imaging evidence of substantial salvageable penumbra are responsive to acute stroke therapies. [10,11] Sites of vessel occlusion will include any cortical vessel occlusion including distal branches of MCA (M3, M4), ACA, PCA, and PICA, in addition to LVO's (ICA, M1, and M2 branches of MCA), and regarding the severity of deficits, we will include NIHSS < 4 in the presence of a disabling deficit, in addition to NIHSS ≥ 4. This patient population with more distal vessel occlusion and less severe deficits are the likely patients not proceeding to thrombectomy even in the current expanded treatment era, and who are also as informative regarding the main safety, tolerability, and feasibility endpoints of the study.

D.3.2.Enrollment and Consent

All acute ischemic stroke patients within 24 hours of their symptoms onset who present to Roland Reagan Medical Center (Emergency Department or inpatient hospital if they are already admitted for a different indication) and meet the study inclusion/exclusion criteria will be identified by a study physician-investigator and subsequently offered enrollment in the trial. Prospective subjects will be provided with written and verbal information regarding the nature of the study, the procedures and evaluations involved, and the potential risks and benefits. All participating patients or their legally authorized representatives (LAR) will provide explicit written informed consent.

Not all adult subjects will have the capacity to give informed consent. The likely range of impairment includes stupor and aphasia. By interviewing the patient, the investigators will assess whether the affected individual understands the central elements of the study procedures and has the capacity for informed consent, using the recommended approach of the institution's Institutional Review Board, such as the UCLA Office of Human Research Protection Decision-Making Capacity Assessment Tool.

Patients with capacity to consent will be invited to participate by the PI. In patients without the capacity to consent, the patient's LAR will be asked to provide consent for participation. If the LAR is not physically available but reachable through the phone at the time of enrollment, the informed consent will be sent to the LAR via fax or email after discussing the details of the study via phone. Then the LAR returns the signed/e-signed form to the PI, again either via tax or email.

The investigator will inform the patient or legally authorized representative of the availability of the study as follows: "You (your relative) is having a stroke. We are doing a research study of a new treatment for stroke. Here is an informed consent form that describes the study. Please read it. After you are finished, I will answer any questions you may have." Once subjects or their legally authorized representatives have read and understood the IRB-approved consent form, and had all their questions answered, written informed consent will be elicited.

D.4. Dose Tiers and Randomization

We will implement a traditional 3+3 (rule-based, modified Fibonacci) dose escalation design, with 3:1 randomization to active treatment vs sham control. There will be 6 dose tiers, reflecting increasing intensity and duration of stimulation: Tier 1 – 1 mA, single 20 - min cycle; Tier 2– 2 mA, single 20 min cycle; Tier 3 – 1 mA, 2 cycles of 20 min/20 min off; Tier 4– 2 mA, 2 cycles of 20 min/20 min off; Tier 5 – 1 mA, 3 cycles of 20 min/20 min off; Tier 6 – 2 mA, 3 cycles of 20 min/20 min off (Figure 7). Patients in the sham stimulation arm at all the tiers will have the cap and electrodes in place, and switches moved but without any delivered electrical stimulation. While the highest dose tier in this study is expected to be fully safe, based on preclinical and clinical studies [12,19], since this is a first-in-human study in acute stroke, a formal escalation to higher dose tiers is prudent.

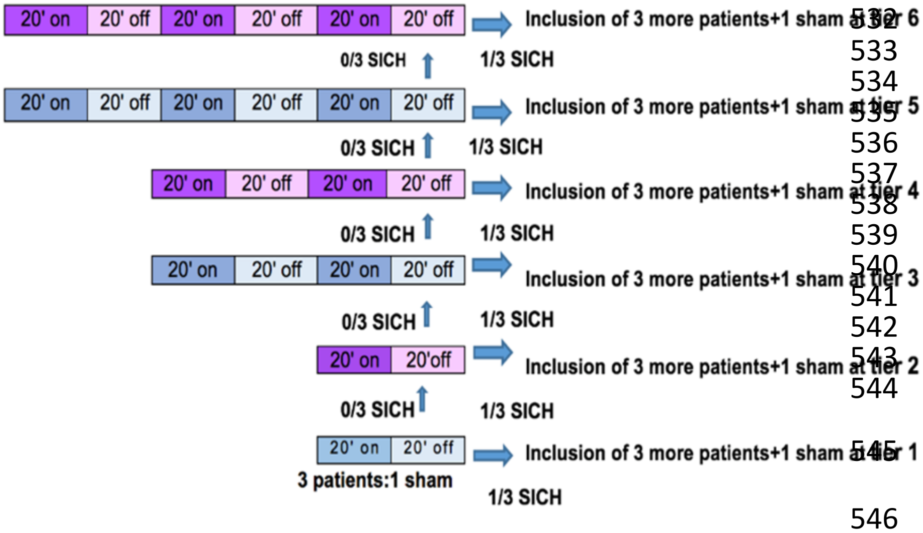

Figure 7. This figure shows the 6 escalating tiers. Blue represents 1 mA and purple shows 2 mA of tDCS. Symptomatic ICH (SICH) in none of the three patients in a tier will result in escalation. However, SICH in one patient, 3 more patients and 1 sham will be enrolled at the same tier.

D.4. Management during and post stimulation period

Figure7: Technician-filled tolerability form	*Presence*		*Severity*			*Effect on the study treatment (Shortened the stim duration)*		*Duration/Frequency*			*Treatment*		
**Adverse Events**	**A**	**P**	**M**	**Mod**	**S**	**Y**	**N**	**< 50 % of stim period**	**≥50% of stim period**	**lasted after stim period (min)**	**None**	**Con**	**Med**
**Rash**													
**Skin dryness**													
**Hair loss**													
**Itching**													
**Hives**													
**Skin Sensitivity to light**													
**Skin soreness**													
**Skin redness**													
**Skin darkening**													
**Numbness**													
**Tingling**													
**Dizziness**													
**General Pain**													
**Headache**													

***A**=Absent, **Con**=Conservative therapy such as cold pack and lotion, **M**= Mild, **Mod**=Moderate, **Med**= Medication such as topical steroid, **N**= No, **P**= Present, **R**: Resolved, **S**: Severe, **Stim**= Stimulation, **Y**=Yes.

Figure 8. Shows the technician-filled tolerability form. The selected items are based on the most common reported adverse effects associated with tDCS.

After randomization and during the stimulation, all patients will be monitored closely by a physician-investigator. NIHSS will be obtained at the end of each 20-minute stimulation cycle and a visual inspection of the skin and rate degree of any potential erythema under the electrode will be performed. Then, a tolerability form will be completed based on validated cutaneous, neurological, and pain items of the PRO-CTAE (Patient-Reported Outcomes version of the Common Terminology Criteria for Adverse Events) (Figure 8). [49,50].

After the stimulation, nursing neurologic assessments will be performed by Neurointensive Care Unit or Stroke Unit nurses with extensive experience in monitoring acute stroke patients. Specifically, patients will be monitored for any signs of neurological worsening and will be queried on development of new or worsening neurological symptoms including headache, dizziness, numbness, or weakness, etc. If any sign or symptom of neurological worsening is suspected, the patient will be immediately assessed by the on-call neurologist and an immediate brain imaging (CT or MRI) will be obtained. Neuro-checks will be performed at 1h and 2h after stimulation and then every 2 hours in patients requiring Neurointensive Care and every 4 hours in patients requiring Stroke Unit Care. Subsequent care will be continued in these settings, including medical management per national guidelines for acute ischemic stroke management issued by the American Stroke Association.[51] Patients will not receive NSAIDs as concomitant therapy for the first 7 days after enrollment. All patients will undergo a multimodal MRI or CT including standard parenchymal images, non-invasive angiography, and perfusion studies and formal NIHSS assessment at 2-hour and 24-hour following the end of the stimulation tier as part of the study protocol.

D.6. Study Visits and Data Acquisition

D.6.1 Schedule of Events

	Baseline	End of each 20-min stimulation cycle			2-hr	24-hr	Day 4	Day 30	Day 90
		1	2	3					
**Consent**	✔								
**History / Exam**	✔								
**Routine Labs**	✔								
**Vital Signs**	✔	✔	✔	✔					
**Patient tolerability form**		✔	✔	✔					
**Technician tolerability form/Skin Inspection***	✔	✔	✔	✔					
**Multimodal MRI or CT**	✔				✔	✔			
**NIHSS**	✔	✔	✔	✔	✔	✔			✔
**Modified Rankin Scale (mRS)**	✔						✔	✔	✔
**Barthel Index (BI)**									✔
**EuroQol (EQ-5D)**									✔
**AMC Linear Disability Scale**									✔
**Interval serious adverse events**		✔	✔	✔	✔	✔	✔	✔	✔
**Concomitant/Interval medications/procedures**	✔	✔	✔	✔	✔	✔	✔	✔	✔

* Skin Inspection will be performed before each stimulation period

D.6.2. Baseline Evaluation

- Demographics (age, sex, race)
- Last known well time
- Past medical/surgical history including vascular diagnoses and risk factors (stroke, TIA, carotid stenosis, myocardial infarction, atrial fibrillation, peripheral arterial disease, hypertension, diabetes, dyslipidemia)
- Medications, including antithrombotics, antihypertensives, statins, anti-arrhythmics Family history of vascular disease;
- Tobacco (including timing, duration, amount), alcohol, and illicit drug use;
- Vital signs (systolic blood pressure, diastolic blood pressure, pulse, respiratory rate, oxygen saturation);
- Premorbid global disability (modified Rankin Scale);
- Neurological deficits severity (NIHSS);
- Laboratory results (CBC, platelet count, glucose, lytes, INR, PTT, LFTs.

D.6.3. During tDCS Treatment

- NIH Stroke Scale (NIHSS),
- Tolerability/AE Form – Technologist, Tolerability Questionnaire – Patient

D.6.4. Early (2 Hours) After tDCS

- NIHSS,
- Multimodal MRI/CT
- Interval serious adverse events
- Interval medications/procedures

D.6.5. Late (24 Hours) After tDCS

- NIHSS
- Multimodal MRI/CT
- Interval serious adverse events
- Interval medications/procedures

D.6.6. Day 4

- Modified Rankin Scale (mRS)
- Interval serious adverse events
- Interval medications/procedures

D.6.7. Day 30

- mRS (phone),
- Interval serious adverse events
- Interval medications/procedures

D.6.8. Day 90

- NIHSS,
- Modified Rankin Scale (mRS)
- AMC Linear Disability Scale (granular disability)
- Barthel Index (BI) (activities of daily living)
- EuroQol (EQ-5D) (health-related quality of life)
- Interval serious adverse events
- Interval medications/procedures

D.6.9. Imaging Assessments

- <u>Baseline</u>

Emergent multimodal MRI or CT is currently acquired as the routine initial imaging study in all acute stroke patients at UCLA without contraindications such as the presence of a pacemaker or metal implant. A 1.5 T or 3 T scanner equipped with echo-planar imaging capability is used for rapid acquisition of diffusion and perfusion scans. The standard clinical MRI protocol includes Gradient Recall- Echo (GRE), DWI, FLAIR (Fluid-Attenuated Recovery Image), PWI and MR angiography. The ADC (apparent -diffusion coefficient) values derived from DWI acquisition (b=0, 1000 s/mm^2 applied in each of three principal gradient directions) will be used to delineate the volume of ischemic core. The tissue with ADC values of ≤ 620 μm^2/s will be considered ischemic core, indicative of tissue with advanced, irreversible bioenergetic compromise. FLAIR delineates early parenchymal signal abnormality associated with ischemia and slow retrograde flow in leptomeningeal collaterals appearing as FLAIR vascular hyperintensity (FVH). The GRE sequence is used to evaluate the presence of intracranial hemorrhage and deoxygenated leptomeningeal collaterals appearing as GRE vascular hypointensity (GVH). PWI is acquired with sequential T2*-weighted (gradient echo) EPI time sequence scanning. Early in the time series, a bolus (0.1 mmol/kg) of MRI contrast material is rapidly infused (5 ml/sec through an 18 or larger gauge angiocatheter) using a power injector. The perfusion lesion volume (tissue at risk volume) will be the region with Tmax ≥ 6sec (Tmax = the time delay from the arrival of contrast to its peak concentration at the tissue vasculature). [52,53] Intracranial CEMRA (Contrast-Enhanced MRA) will identify large and medium vessel occlusion.

In patients receiving CT, the standard clinical CT protocol includes non-contrast CT (NCCT), multiphase CT angiography, CT perfusion (CTP) processed through RAPID software. NCCT is used to rule out acute intracranial hemorrhage appearing as hyperdensity. The tissue with rCBF < 30% on CTP will be considered ischemic core and the perfusion lesion volume (tissue at risk volume) will be the region with Tmax ≥ 6sec. CT angiography (CTA) will identify large and medium vessel occlusion.

- <u>2h and 24h Post-Stimulation</u>

Follow-up MR or CT imaging will be obtained 2h and 24h post-stimulation (supported by Radiology Dept. research funds). MRI Imaging sequences will include DWI/FLAIR/GRE/PWI/CEMRA and CT imaging sequences will consist of NCCT/CTA/CTP. RAPID image processing software will be applied to the above images to quantify, at the baseline, 2h, and 24h time-points: 1) Ischemic Core volume, 2) Perfusion lesion volume, and 3) Penumbra volume (perfusion volume - core volume). From these values, the following measures will be constructed: 1) Early penumbra preservation: Volume of baseline penumbra tissue not progressing to ischemic core at 2h; 2) Penumbral salvage: Volume of baseline penumbra tissue not progressing to ischemic core at 24h; 3) Early collateral flow enhancement: Perfusion lesion volume at baseline – Perfusion lesion volume at 2h; 4) Sustained collateral flow enhancement: Perfusion lesion volume at baseline – Perfusion lesion volume at 24h; 5) Early infarct growth: Ischemic core lesion volume at 2h – Ischemic core lesion volume at baseline; and 6) Final infarct growth: Ischemic core lesion volume at 24h – Ischemic core lesion volume at baseline. Additional location and extent of ischemic injury at baseline, 2h, and 24h will be rated using the MRI ASPECTS scoring system, with regions considered involved if diffusion restriction is present in more than 20% of the region or CT ASPCETS. [54,55] In patients receiving MRI, location and extent of FVH and GVH will be rated using the FVH-modified ASPECTS and GVH modified ASPECTS scales.[56]

D.6.10 Adverse Events

D.6.10.1 Serious Adverse Events (SAEs)

All serious adverse events occurring during the 90 days of study participation will be recorded. A serious adverse event is any adverse event that is fatal, is life-threatening, is permanently or substantially disabling, requires or prolongs hospitalization, or requires medical or surgical intervention to prevent one of the above outcomes.[19]

D.6.10.2 Symptomatic intracranial hemorrhage

The lead safety endpoint adverse event is symptomatic intracranial hemorrhage (SICH), defined using the SWIFT PRIME trial criteria [55]: an increase of 4 or more points on the NIHSS within 24 hours of stimulation associated with parenchymal hematoma type 1 (PH1), parenchymal hematoma type 2 (PH2), remote intraparenchymal hemorrhage (RIH), subarachnoid hemorrhage (SAH), or intraventricular hemorrhage (IVH). In addition, all hemorrhages, both symptomatic and asymptomatic, will also be separately classified and analyzed by radiologic subtype, as hemorrhagic infarction type 1 (HI1), hemorrhagic infarction type 2 (HI2), PH1, PH2, RIH, SAH, or IVH.[55] A central neuroimaging core lab, blinded to treatment assignment, will review all brain MRI scans obtained at 24h and rate presence and type of radiologic hemorrhagic transformation. In addition, in patients who experienced worsening by 4 or more NIHSS points in the first 24 hours, they will review any and all additional brain MRI or CT scans obtained during the 24h time period,

SICH is the primary safety endpoint of the current trial, but trial results will be assessed on a variety of additional safety, feasibility, and tolerability results as well. It is anticipated that, after completion of the current trial, the judgement of whether to proceed directly to a pivotal trial, to proceed to a larger safety and preliminary efficacy trial, or to not proceed with further development, will rest on a considered and informed assessment of all outcome measures. It is important therefore to collect data on a wide range of safety endpoints and a wide range of measures of functional outcome, as is

planned in this proposal. In making the selection of a primary safety endpoint, an emphasis was placed on ensuring patient safety throughout the course of the trial by choosing an endpoint with uncontestable clinical relevance (Symptomatic Intracranial Hemorrhage).

D.6.10.3 Additional Adverse Events with Specific Interrogation

In addition to general screening for all serious adverse events and focused elicitation of symptomatic intracranial hemorrhage events, the following events will be specifically interrogated for and recorded in the case report forms: skin redness, scalp rash, hair loss, seizure, headache, sensitivity to light, new ischemic stroke, deep venous thrombosis, pulmonary embolism, pneumonia, acute MI.

E. Safety Monitoring

E.1. Data and Safety Monitoring Plan

The trial will be monitored by an Independent Data and Safety Monitoring Board (DSMB). Dr. Bruce Dobkins from department of neurorehabilitation will lead the DSMB. DSMB will assess for the causal relationship of the serious adverse event to the study treatment as definite, probable, possible, unlikely, or unrelated.

DSMB will meet at the completion of each dose tier, review all safety data, and determine whether the study will proceed to the next dose tier. DSMB deliberations will be guided by: 1) a formal stopping/escalation rule, based on the occurrence of the lead safety endpoint, symptomatic intracranial hemorrhage (SICH), and 2) The DSMB members' clinical judgement upon review of all other safety outcomes. In addition, DSMB will monitor separately the patients who enroll in the study with a stroke scale 0-3 during the study as they are treated to determine if they have an increase in NIHSS of 2 or more or which is disabling.

Furthermore, Food and Drug Administration (FDA) will be notified if there is one occurrence of SICH prior to repeating the tier or escalating to a higher tier.

E. 2. Statistical Design and Analyses Plan

E.2.1. Sample size

The study sample size derives from the use of the 3+3, rule-based, modified Fibonacci, dose escalation design, with 3:1 randomization to active treatment vs sham control. There will be 6 dose tiers, reflecting increasing intensity and duration of stimulation (Figure 7). The 3+3 study design (3 patients and 1 sham) with 6 dose tiers yields a sample size of at least 24 and potentially up to 48. The 3+3 design is the classical approach to dose escalation in first-in-human studies. [57,58] While newer, adaptive designs for dose escalation trials have been developed, they are more complex and have limited advantages when major toxicities are not expected. [59] Therefore, the rule-based, 3+3 design remains the dominantly employed approach in current dose-escalation studies. We will take every step to avoid missing data by scheduling of the follow-up visits early in visit time windows and readiness to travel to the patient's location to perform needed assessments. Should any missing data occur, primary analyses will be performed using multiple imputation, with sensitivity analyses including complete case and worst possible outcome analyses. [60]

E.2.2. Baseline characteristics

The demographic and baseline clinical characteristics of the study population will be delineated with standard descriptive statistics. Categorical variables describing the clinical history, examination findings, and initial treatment will be summarized by

frequencies. Continuous variables such as vital signs, laboratory results, and time variables will be characterized by means, standard deviations, and 95% confidence intervals (CI). Ordinal and non-normally distributed variables (such as the NIHSS) will be characterized by medians and interquartile ranges. Baseline characteristics will be compared between the tDCS stimulation group with sham group to assess covariate balance. Wilcoxon Rank-Sum tests will be used for continuous or ordinal variables; Fisher's exact tests and chi-square tests will be used for grouped or nominal categorical variables.

E.2.3. Safety and Tolerability (Specific Aim 1)

E.2.3.1 Dose Escalation

The study Data and Safety Monitoring Board (DSMB) will meet at the completion of each dose tier, review all safety data, and determine whether the study will proceed to the next dose tier. DSMB deliberations will be guided by: 1) a formal stopping/escalation rule, based on the occurrence of the lead safety endpoint, symptomatic intracranial hemorrhage (SICH), and 2) The DSMB members' clinical judgement upon review of all other safety outcomes. The formal dose escalation rule uses SICH frequency to gate the occurrence and pace of escalation through the 6 dose tiers. If no SICH occurs in the 3 active patients at a dose tier, enrollment may escalate to the next dose tier. If one SICH occurs, 3 more active (and 1 more control) patient will be enrolled at that dose tier before escalation. If 2 SICHs occur at a dose tier, further study enrollment is held until detailed review by the DSMB (Fig 6). In addition, whenever the formal SICH criteria for dose escalation has been met, the DSMB will formally meet, review the SICH data and all other safety data, and advice regarding proceeding to the next tier, continuing at the current tier, or placing the study on hold.

E.2.3.2. Primary Safety Endpoint Analysis

For the final statistical analysis of the primary SICH safety endpoint, a chi-square test will be used to detect differences in the rate of SICH between the active treatment and sham patients and higher and lower dose tiers. The treatment will be considered to have exhibited adequate safety in the current trial to proceed to future, larger, pivotal efficacy trials if tDCS results in lower or equivalent rates of SICH compared to sham.

E.2.3.3. Secondary Safety Endpoints Analysis

In secondary safety endpoint analyses, the following will be compared between the active treatment and sham patients, and between higher and lower dose tiers, using chi-squared tests: **1)** Asymptomatic ICH by 24h (intracranial hemorrhage not associated with NIHSS worsening ≥ 4); **2)** Early neurologic deterioration (worsening ≥ 4 on NIHSS during the 24-hour period after stimulation, with or without intracranial hemorrhage); **3)** All-cause mortality at day 90 (mRS); and **4)** All serious adverse events.

E.3.3. Tolerability Endpoint Analysis

The lead tolerability endpoint is completion of the protocol-assigned stimulation treatment without early cessation due to cutaneous, neurologic, nociceptive or other adverse effects. Experience with tDCS in post-stroke patients indicates only infrequent cutaneous (itching, tingling) adverse effects are likely to occur. Accordingly, for the current study, a patient will be considered to have tolerated the procedure if at least 75% of the stimulus period was completed. The treatment will be considered generally tolerable if, among all enrolled patients, tolerated procedures are achieved in ≥90% of patients, assessed with a one-sided p value of 0.025. Secondary tolerability endpoints

will be the rate and severity of cutaneous, neurological, and pain items of the technician-filled (Figure 7) and patient-filled tolerability forms [49], descriptively compared between active treatment and sham patients, and between higher and lower dose tiers.

E.3.4. Feasibility Endpoint Analysis (Specific Aim 2)

A time-motion analysis and mock run-throughs will be conducted prior to first enrollment. Nonetheless, it is anticipated that processes to optimize rapid placement of the cap and electrodes will continue to improve with experience gained from initially enrolled patients. The predefined success threshold for feasibility will be median time from randomization to tDCS initiation ≤ 10 minutes in the last 10 enrolled patients.

E.3.5. Exploratory Efficacy Endpoints Analysis (Specific Aim 3)

E.3.5.1. Exploratory Imaging Biomarker Efficacy Endpoints

This study is underpowered to definitively determine efficacy, so all imaging efficacy analyses will be purely exploratory and descriptive. Imaging biomarker efficacy endpoints will be characterized in the active and sham patients, and in higher and lower dose tiers, using means and 95% confidence intervals. The six imaging efficacy endpoints of greatest interest that will be explored are: early and late penumbral salvage, early and late collateral flow enhancement, and early and late infarct growth.

E.3.5.2. Exploratory Clinical Efficacy Endpoints

This study is underpowered to definitively determine efficacy, so all clinical efficacy analyses will be purely exploratory and descriptive. Four clinical outcome measures were selected based on their reliability, familiarity to the neurologic community, and adaptability for use in patients who have had a stroke. These endpoints are: the modified Rankin Scale (mRS), a rating of global disability; the Barthel Index (BI), a measure of instrumental activities of daily living; the National Institutes of Health Stroke Scale (NIHSS), a measure of neurologic deficit severity; and the EuroQol (EQ-5D), an assessment of health-related quality of life; and AMC Linear Disability Scale, a granular degree of disability.

Clinical efficacy endpoints will be characterized in the active and sham patients, and in higher and lower dose tiers. Early course clinical efficacy endpoints of greatest interest that will be explored are: **1)** Normalized change in neurologic deficit from baseline to 24h (normalized delta NIHSS – linear variable, analyzed with means and 95% CIs; and **2)** Degree of neurologic deficit at 24h (NIHSS – quasi-linear variable, analyzed with means and 95% CIs). Final outcome clinical efficacy endpoints of greatest interest that will be explored are: **1)** Degree of disability at 90 days, assessed across all 7 levels of the modified Rankin Scale – ordinal variable, analyzed with medians (IQRs) and means (95% CIs); **2)** Functional independence (mRS 0-2) at 90 days – binary variable, analyzed with rates and 95% CIs; **3)** Granular degree of disability at 90 days (AMC Linear Disability Scale) – linear variable, analyzed with means and 95% CIs; and **4)** Health-related quality of life (EQ-5D) – linear variable, analyzed with means and 95% CIs.

References:

1. Benjamin EJ, Blaha MJ, Chiuve SE, et al. Heart Disease and Stroke Statistics-2017 Update: A Report From the American Heart Association. *Circulation.* 2017.
2. Campbell BC, Donnan GA, Lees KR, et al. Endovascular stent thrombectomy: the new standard of care for large vessel ischaemic stroke. *Lancet Neurol.* 2015;14(8):846-854.
3. Adeoye O, Hornung R, Khatri P, Kleindorfer D. Recombinant tissue-type plasminogen activator use for ischemic stroke in the United States: a doubling of treatment rates over the course of 5 years. *Stroke.* 2011;42(7):1952-1955.
4. Saver JL, Goyal M, van der Lugt A, et al. Time to Treatment With Endovascular Thrombectomy and Outcomes From Ischemic Stroke: A Meta-analysis. *JAMA.* 2016;316(12):1279-1288.
5. Haussen DC, Nogueira RG, Elhammady MS, et al. Infarct growth despite full reperfusion in endovascular therapy for acute ischemic stroke. *J Neurointerv Surg.* 2016;8(2):117-121.
6. Goyal M, Menon BK, van Zwam WH, et al. Endovascular thrombectomy after large-vessel ischaemic stroke: a meta-analysis of individual patient data from five randomised trials. *Lancet.* 2016;387(10029):1723-1731.
7. Tymianski M. Novel approaches to neuroprotection trials in acute ischemic stroke. *Stroke.* 2013;44(10):2942-2950.
8. Saver JL, Starkman S, Eckstein M, et al. Prehospital use of magnesium sulfate as neuroprotection in acute stroke. *N Engl J Med.* 2015;372(6):528-536.
9. Warach S, Pettigrew LC, Dashe JF, et al. Effect of citicoline on ischemic lesions as measured by diffusion-weighted magnetic resonance imaging. Citicoline 010 Investigators. *Ann Neurol.* 2000;48(5):713-722.
10. Albers WG. Endovascular Therapy Following Imaging Evaluation for Ischemic Stroke 3 (DEFUSE 3). *https://clinicaltrialsgov/ct2/show/NCT02586415*. 2017.
11. Nogueira RG, Jadhav AP, Haussen DC, et al. Thrombectomy 6 to 24 Hours after Stroke with a Mismatch between Deficit and Infarct. *N Engl J Med.* 2017.
12. Woods AJ, Antal A, Bikson M, et al. A technical guide to tDCS, and related non-invasive brain stimulation tools. *Clin Neurophysiol.* 2016;127(2):1031-1048.
13. Nitsche MA, Seeber A, Frommann K, et al. Modulating parameters of excitability during and after transcranial direct current stimulation of the human motor cortex. *J Physiol.* 2005;568(Pt 1):291-303.
14. Nitsche MA, Nitsche MS, Klein CC, Tergau F, Rothwell JC, Paulus W. Level of action of cathodal DC polarisation induced inhibition of the human motor cortex. *Clin Neurophysiol.* 2003;114(4):600-604.

15. Brunoni AR, Nitsche MA, Bolognini N, et al. Clinical research with transcranial direct current stimulation (tDCS): challenges and future directions. *Brain Stimul.* 2012;5(3):175-195.
16. Dmochowski JP, Datta A, Huang Y, et al. Targeted transcranial direct current stimulation for rehabilitation after stroke. *Neuroimage.* 2013;75:12-19.
17. Teichmann M, Lesoil C, Godard J, et al. Direct current stimulation over the anterior temporal areas boosts semantic processing in primary progressive aphasia. *Ann Neurol.* 2016;80(5):693-707.
18. Wessel MJ, Zimerman M, Hummel FC. Non-invasive brain stimulation: an interventional tool for enhancing behavioral training after stroke. *Front Hum Neurosci.* 2015;9:265.
19. Bikson M, Grossman P, Thomas C, et al. Safety of Transcranial Direct Current Stimulation: Evidence Based Update 2016. *Brain Stimul.* 2016;9(5):641-661.
20. Chhatbar PY, Chen R, Deardorff R, et al. Safety and tolerability of transcranial direct current stimulation to stroke patients - A phase I current escalation study. *Brain Stimul.* 2017;10(3):553-559.
21. Harukuni I, Bhardwaj A. Mechanisms of brain injury after global cerebral ischemia. *Neurol Clin.* 2006;24(1):1-21.
22. Nakamura H, Strong AJ, Dohmen C, et al. Spreading depolarizations cycle around and enlarge focal ischaemic brain lesions. *Brain.* 2010;133(Pt 7):1994-2006.
23. Hossmann KA. Periinfarct depolarizations. *Cerebrovasc Brain Metab Rev.* 1996;8(3):195-208.
24. Hartings JA, Rolli ML, Lu XC, Tortella FC. Delayed secondary phase of peri-infarct depolarizations after focal cerebral ischemia: relation to infarct growth and neuroprotection. *J Neurosci.* 2003;23(37):11602-11610.
25. Notturno F, Pace M, Zappasodi F, Cam E, Bassetti CL, Uncini A. Neuroprotective effect of cathodal transcranial direct current stimulation in a rat stroke model. *J Neurol Sci.* 2014;342(1-2):146-151.
26. Peruzzotti-Jametti L, Cambiaghi M, Bacigaluppi M, et al. Safety and efficacy of transcranial direct current stimulation in acute experimental ischemic stroke. *Stroke.* 2013;44(11):3166-3174.
27. Baba T, Kameda M, Yasuhara T, et al. Electrical stimulation of the cerebral cortex exerts antiapoptotic, angiogenic, and anti-inflammatory effects in ischemic stroke rats through phosphoinositide 3-kinase/Akt signaling pathway. *Stroke.* 2009;40(11):e598-605.
28. Kim SJ, Kim BK, Ko YJ, Bang MS, Kim MH, Han TR. Functional and histologic changes after repeated transcranial direct current stimulation in rat stroke model. *J Korean Med Sci.* 2010;25(10):1499-1505.
29. Fox JL, Yasargil MG. The experimental effect of direct electrical current on intracranial arteries and the blood-brain barrier. *J Neurosurg.* 1974;41(5):582-589.
30. Iacoboni M. The role of premotor cortex in speech perception: evidence from fMRI and rTMS. *J Physiol Paris.* 2008;102(1-3):31-34.
31. Aziz-Zadeh L, Iacoboni M, Zaidel E, Wilson S, Mazziotta J. Left hemisphere motor facilitation in response to manual action sounds. *Eur J Neurosci.* 2004;19(9):2609-2612.
32. Iacoboni M. Visuo-motor integration and control in the human posterior parietal cortex: evidence from TMS and fMRI. *Neuropsychologia.* 2006;44(13):2691-2699.

33. Dunn W, Rassovsky Y, Wynn JK, et al. Modulation of neurophysiological auditory processing measures by bilateral transcranial direct current stimulation in schizophrenia. *Schizophr Res.* 2016;174(1-3):189-191.
34. Rassovsky Y, Dunn W, Wynn J, et al. The effect of transcranial direct current stimulation on social cognition in schizophrenia: A preliminary study. *Schizophr Res.* 2015;165(2-3):171-174.
35. Dunn W, Rassovsky Y, Wynn J, et al. The effect of bilateral transcranial direct current stimulation on early auditory processing in schizophrenia: a preliminary study. *J Neural Transm (Vienna).* 2017.
36. Reis DJ, Berger SB, Underwood MD, Khayata M. Electrical stimulation of cerebellar fastigial nucleus reduces ischemic infarction elicited by middle cerebral artery occlusion in rat. *J Cereb Blood Flow Metab.* 1991;11(5):810-818.
37. Reis DJ, Kobylarz K, Yamamoto S, Golanov EV. Brief electrical stimulation of cerebellar fastigial nucleus conditions long-lasting salvage from focal cerebral ischemia: conditioned central neurogenic neuroprotection. *Brain Res.* 1998;780(1):161-165.
38. Yamamoto S, Golanov EV, Reis DJ. Reductions in focal ischemic infarctions elicited from cerebellar fastigial nucleus do not result from elevations in cerebral blood flow. *J Cereb Blood Flow Metab.* 1993;13(6):1020-1024.
39. Zhou P, Qian L, Glickstein SB, Golanov EV, Pickel VM, Reis DJ. Electrical stimulation of cerebellar fastigial nucleus protects rat brain, in vitro, from staurosporine-induced apoptosis. *J Neurochem.* 2001;79(2):328-338.
40. Glickstein SB, Ilch CP, Reis DJ, Golanov EV. Stimulation of the subthalamic vasodilator area and fastigial nucleus independently protects the brain against focal ischemia. *Brain Res.* 2001;912(1):47-59.
41. Bikson M. The coupled vascular hypothesis for transcranial direct current stimulation (tDCS). In. Unpublished work.2017.
42. Samdani AF, Dawson TM, Dawson VL. Nitric oxide synthase in models of focal ischemia. *Stroke.* 1997;28(6):1283-1288.
43. Huisa BN, Stemer AB, Walker MG, et al. Transcranial laser therapy for acute ischemic stroke: a pooled analysis of NEST-1 and NEST-2. *Int J Stroke.* 2013;8(5):315-320.
44. Shuaib A, Bornstein NM, Diener HC, et al. Partial aortic occlusion for cerebral perfusion augmentation: safety and efficacy of NeuroFlo in Acute Ischemic Stroke trial. *Stroke.* 2011;42(6):1680-1690.
45. Bahr Hosseini M WG, Hinman JD, Sharma LK, Rao NM, Starkman S, Yoo B, Scalzo F, Liebeskind DS, Saver JL,for the UCLA Penumbral Imaging Investigators. Prevalence of substantial salvageable penumbra in patients with large vessel occlusion who do not undergo endovascular therapy. In. *European Stroke Organisation Conference (ESOC)*2017.
46. Butcher KS, Parsons M, MacGregor L, et al. Refining the perfusion-diffusion mismatch hypothesis. *Stroke.* 2005;36(6):1153-1159.
47. Furlan AJ, Eyding D, Albers GW, et al. Dose Escalation of Desmoteplase for Acute Ischemic Stroke (DEDAS): evidence of safety and efficacy 3 to 9 hours after stroke onset. *Stroke.* 2006;37(5):1227-1231.

48. Hacke W, Albers G, Al-Rawi Y, et al. The Desmoteplase in Acute Ischemic Stroke Trial (DIAS): a phase II MRI-based 9-hour window acute stroke thrombolysis trial with intravenous desmoteplase. *Stroke.* 2005;36(1):66-73.
49. Basch E, Reeve BB, Mitchell SA, et al. Development of the National Cancer Institute's patient-reported outcomes version of the common terminology criteria for adverse events (PRO-CTCAE). *J Natl Cancer Inst.* 2014;106(9).
50. Dueck AC, Mendoza TR, Mitchell SA, et al. Validity and Reliability of the US National Cancer Institute's Patient-Reported Outcomes Version of the Common Terminology Criteria for Adverse Events (PRO-CTCAE). *JAMA Oncol.* 2015;1(8):1051-1059.
51. Powers WJ, Rabinstein AA, Ackerson T, et al. 2018 Guidelines for the Early Management of Patients With Acute Ischemic Stroke: A Guideline for Healthcare Professionals From the American Heart Association/American Stroke Association. *Stroke.* 2018;49(3):e46-e110.
52. Albers GW, Lansberg MG, Kemp S, et al. A multicenter randomized controlled trial of endovascular therapy following imaging evaluation for ischemic stroke (DEFUSE 3). *Int J Stroke.* 2017;12(8):896-905.
53. Bahr Hosseini M, Liebeskind DS. The role of neuroimaging in elucidating the pathophysiology of cerebral ischemia. *Neuropharmacology.* 2017.
54. Demchuk AM, Coutts SB. Alberta Stroke Program Early CT Score in acute stroke triage. *Neuroimaging Clin N Am.* 2005;15(2):409-419, xii.
55. Saver JL, Goyal M, Bonafe A, et al. Stent-retriever thrombectomy after intravenous t-PA vs. t-PA alone in stroke. *N Engl J Med.* 2015;372(24):2285-2295.
56. Mahdjoub E, Turc G, Legrand L, et al. Do Fluid-Attenuated Inversion Recovery Vascular Hyperintensities Represent Good Collaterals before Reperfusion Therapy? *AJNR Am J Neuroradiol.* 2017.
57. Hansen AR, Graham DM, Pond GR, Siu LL. Phase 1 trial design: is 3 + 3 the best? *Cancer Control.* 2014;21(3):200-208.
58. Penel N, Isambert N, Leblond P, Ferte C, Duhamel A, Bonneterre J. "Classical 3 + 3 design" versus "accelerated titration designs": analysis of 270 phase 1 trials investigating anti-cancer agents. *Invest New Drugs.* 2009;27(6):552-556.
59. Le Tourneau C, Lee JJ, Siu LL. Dose escalation methods in phase I cancer clinical trials. *J Natl Cancer Inst.* 2009;101(10):708-720.
60. Little RJ, D'Agostino R, Cohen ML, et al. The prevention and treatment of missing data in clinical trials. *N Engl J Med.* 2012;367(14):1355-1360.
