# Supplementary material for: High-definition Cathodal Direct Current Stimulation for Treatment of Acute Ischemic Stroke: A Randomized Clinical Trial
Source: JAMA Netw Open. 2023 Jun 21;6(6):e2319231. doi: 10.1001/jamanetworkopen.2023.19231 (PMC10285579; doi:10.1001/jamanetworkopen.2023.19231)
Supplement: Supplement 2. — eMethods. eTable 1. Entry Criteria eTable 2. Reasons for Patients' Ineligibility for Intravenous Thrombolysis and Endovascular Thrombectomy eTable 3. Demographic and Clinical Patient Features at Baseline eTable 4. Main Outcomes Analysis eTable 5. Per Protocol Exploratory Imaging Analysis eTable 6. Per Protocol Exploratory Clinical Analysis [file jamanetwopen-e2319231-s002.pdf]

Supplemental Online Content

Bahr-Hosseini M, Nael K, Unal G, et al; for the TESSERACT Trial Group. High-definition cathodal direct current stimulation for treatment of acute ischemic stroke: a randomized clinical trial. *JAMA Netw Open.* 2023;6(6):e2319231. doi:10.1001/ jamanetworkopen.2023.19231

eMethods.

eTable 1. Entry Criteria

eTable 2. Reasons for Patients' Ineligibility for Intravenous Thrombolysis and Endovascular Thrombectomy

eTable 3. Demographic and Clinical Patient Features at Baseline

eTable 4. Main Outcomes Analysis

eTable 5. Per Protocol Exploratory Imaging Analysis

eTable 6. Per Protocol Exploratory Clinical Analysis

This supplemental material has been provided by the authors to give readers additional information about their work.

© 2023 Bahr-Hosseini M et al. *JAMA Network Open.*

eMethods

Study Design:

The study design was a traditional 3+3 (rule-based, modified Fibonacci) dose-escalation, with 3:1 randomization to active treatment vs sham control. The study was triple blinded with patients, treatment team, and outcome assessors blinded to the study treatment. A random allocation sequence table was generated by Biostatistics Core at UCLA using the random number generator in the R software version 4.0.5 (R foundation for statistical computing, Vienna Austria). The interactive web response system (REDCap) was used for allocation concealment and final randomization at the time of patients' enrollment. Per the 3+3 study design, a total enrollment of 24-48 patients and a total of 6 dose tiers were initially planned, increasing in intensity or duration of stimulation: Tier 1 – 1 mA, single 20 - min cycle; Tier 2– 2 mA, single 20 min cycle; Tier 3 – 1 mA, 2 cycles of 20 min/20 min off; Tier 4– 2 mA, 2 cycles of 20 min/20 min off; Tier 5 – 1 mA, 3 cycles of 20 min/20 min off; Tier 6 – 2 mA, 3 cycles of 20 min/20 min off. Occurrence of symptomatic intracranial hemorrhage (SICH) determined the escalation pace. Dose escalation decisions were made in tandem with Data and Safety Monitoring Committee. The escalation guiding rules were: 1) absence of SICH in any of the three active patients at a given tier – escalate; 2) SICH in 1 of 3 active patients – enroll 4 additional patients (3 active, 1 sham) at that tier, escalate if no further SICH; 3) SICH in a 2nd patient at a dose tier – trial stops. Patients in the sham stimulation arm had the cap and electrodes in place, but without any delivered electrical stimulation. A trained HD-tDCS technician who was not part of the care team performed the randomization and the study stimulation. Key entry criteria were patients with acute ischemic stroke within 24hr from onset; imaging evidence of cortical ischemia; presence of salvageable penumbra; and ineligibility for reperfusion therapies (intravenous lytics and endovascular thrombectomy) (full entry criteria are available in Supplement Table 1). The rates of SICH at 24 hours and all serious adverse events were recorded for safety outcomes. The time from randomization to study stimulation initiation was recorded for the feasibility outcome. After randomization and during the stimulation, patients were monitored closely by the physician-investigator. The National Institute of Health Stroke Scale (NIHSS), a validated quantitative assessment tool to measure stroke-related neurological deficit, was obtained at the end of 20-minute stimulation cycle. Immediately following the stimulation cycle, a visual inspection of the skin and completion of a tolerability form based on validated cutaneous, neurological, and pain items of the PRO-CTAE (Patient-Reported Outcomes version of the Common Terminology Criteria for Adverse Events) were performed. The rate of patients completing the study stimulation period was recorded for tolerability outcome.

After the stimulation, patients were monitored in the Neurointensive Care Unit or Stroke Unit, and frequent neurologic assessment was performed by nurses with extensive experience in monitoring acute stroke patients. Specifically, patients were monitored for any signs of neurological worsening and development of

© 2023 Bahr-Hosseini M et al. *JAMA Network Open.*

new or worsening neurological symptoms. Subsequent care was continued in these settings, including medical management per national guidelines for acute ischemic stroke management issued by the American Stroke Association. Patients underwent multimodal MRI or CT at 2-4 hour (Early time point) and 24-30 hour (Late time point) following the end of the stimulation tier. Long-term clinical outcome measure was assessed using the modified Rankin Scale (mRS) of global disability at 90 days post-stimulation. The mRS assesses disability in stroke patients with score of 0 indicating no symptoms, 1 no significant disability despite mild residual symptom, 2 slight disability, 3 moderate disability but able to walk without assistance, 4 moderately severe disability needing assistance for walking, 5 disability requiring constant care for all needs; 6 death. As recruitment was slower than expected, related to the COVID pandemic, the study was stopped after enrollment of the 10th patients (full study protocol is available in Appendix).

Computational Modeling of the Electrical Fields:

The direct current flow models of various 4x 1 high-definition electrode montages were generated on a health "standard" head using HD-Explore software (version 3.2, Soterix Medical Inc, New York). Six current flow patterns that matched the 6 commonly compromised vascular territories: 1) middle cerebral artery (MCA) trunk; 2) MCA superior division; 3) MCA inferior division; 4) posterior cerebral artery; 5) anterior cerebral artery; and 6) posterior inferior cerebellar artery. Following patient randomization, the appropriate montage with current flow pattern visually matching the location of hypoperfused ischemic region on MR or CT perfusion parametric map was selected. The following isotropic direct current electrical conductivities in (S m^{-1}) were assigned: scalp (0.465), skull (0.01), CSF (1.65), gray matter (0.276), white matter (0.126), air (1e-7) electrodes (5.8e7), gel (0.3)[1].

Imaging Analysis:

Imaging analyses were performed by a board-certified neuroradiologist with 10 years of experience who was blinded to the study treatment. MRI Imaging sequences included diffusion-weighted image (DWI)/ fluid-attenuated inversion recovery (FLAIR)/gradient recall echo (GRE)/ Dynamic susceptibility contrast (DSC) perfusion /contrast-enhanced MR angiography. CT imaging sequences consisted of non-contrast CT/CT angiography/CT perfusion. Clinical RAPID image processing software were applied to the above images to quantify, at the baseline, 2h (Early time point), and 24h (Late time-point): 1) Ischemic Core volume (volume of MRI-DWI lesion, relative cerebral blood flow <30% on CTP or hypodensity on NCCT), 2) Hypoperfusion lesion volume (ischemic lesion with time-to-maximum (Tmax)>6sec on DSC-MRI and CTP), 3) Penumbra volume (perfusion volume - core volume). From these values, the following measures were constructed: 1) Early infarct core growth: Early infarct core volume - initial core volume; 2) Total infarct core growth: Late infarct core volume- initial core volume; 3) Early penumbra tissue proportion not

advanced to ischemic core: 1 – [(Early infarct core vol– Initial core vol)/ Initial penumbra vol]; 4) Total Penumbral salvage proportion: 1 - [(Late infarct core vol – Initial core vol)/ Initial penumbra vol]; 5) Early hypoperfusion lesion volume change: Early hypoperfusion lesion volume- Initial hypoperfusion lesion volume; 6) Total hypoperfusion lesion volume change: Late hypoperfusion lesion volume- Initial hypoperfusion lesion volume.

Additional post-processing of early DSC-MRI and CTP images were performed for quantitative relative cerebral blood volume (qrCBV) analyses using Food and Drug Administration–approved software (Olea Sphere SP23; Olea Medical SAS, La Ciotat, France). Perfusion parametric maps of Tmax and rCBV were coregistered between the two scans using a 6-degree-of-freedom transformation and a mutual information cost function. Subsequently, a volume of interest (VOI) was generated from the visually perceptible perfusion abnormality on baseline Tmax maps, which was then automatically transferred over the coregistered follow-up perfusion maps. The CBV values within this VOI on baseline and follow-up scans were obtained using a voxel-based analysis. Finally, the CBV values were normalized to a region of interest placed in the contralateral centrum semiovale and mean rCBV was calculated for each scan.

References:

1. Datta A, Truong D, Minhas P, Parra LC, Bikson M. Inter-Individual Variation during Transcranial Direct Current Stimulation and Normalization of Dose Using MRI-Derived Computational Models. *Front Psychiatry*. 2012;3:91.

eTable 1. Entry Criteria

**Inclusion Criteria**
1. Patient age ≥ 18
2. Last known well-time within 24 hours of randomization
3. NIHSS ≥ 4
4. NIHSS <4 and disabling stroke (a deficit that, if unchanged, would prevent the patient from performing basic activities of daily living such as bathing, ambulating, toileting, hygiene, and eating or returning to work)
5. Presence of any cortical vessel occlusion including internal carotid artery, branches of Middle cerebral artery (MCA), Anterior Cerebral artery (ACA), Posterior Cerebral artery (PCA), Posterior-Inferior cerebellar artery (PICA)
6. Substantial salvageable penumbra (hypoperfusion lesion (Tmax>6sec)/core (ADC≤ 620 $\mu m^2/s$ or CBF <30%) ≥ 1.2)
7. Ineligible for IV tPA per national AHA/ASA Guidelines
8. Ineligible for endovascular thrombectomy per national AHA/ASA Guidelines
9. Informed consent obtained
**Exclusion Criteria**
1. Acute intracranial hemorrhage
2. Ischemic core volume (ADC≤ 620 $\mu m^2/s$) ≥ 100 ml
3. tDCS contraindications - electrically activated metal or non-metal intracranial implants
4. Severe MR contrast allergy or renal dysfunction with eGFR<30ml/min, precluding contrast
5. Pregnancy
6. Signs, symptoms, or EKG evidence of acute myocardial infarction
7. Suspicion of aortic dissection
8. History of seizure disorder or new seizures with presentation of current stroke
9. Any other major life-threatening or serious medical condition that would prevent completion of the study protocol including attendance at the 3-month follow-up visit
10. Already participating in another research therapy treatment trial
11. Skull defect, or scalp lesion at site of stimulation
12. Preexisting coagulopathy, consist of platelet count of ≤ 100, INR ≥ 3, PTT ≥ 90 [a]
13. Patients suspected of having infective endocarditis and ischemic stroke related to septic emboli [b]
14. Patients suspected or known to be infected with coronavirus 2019 (COVID-19) [b]
15. Radiographic evidence or suspicion of chronic conditions that may predispose them to intracranial hemorrhage including brain arteriovenous malformations, cerebral cavernous malformations, cerebral telangiectasia, multiple previous intracerebral hemorrhages (amyloid angiopathy) [b]
16. Suspected cerebral vasculitis based on medical history and CTA/ MRA [b]
17. Suspected cysticercosis [b]
18. Suspected cranial dural arteriovenous fistula [b]

© 2023 Bahr-Hosseini M et al. *JAMA Network Open.*

19. Cerebral venous thrombosis [b]
20. Head trauma causing loss of consciousness, concussion, confusion, or a headache within the past 30 days [b]
21. Hemorrhagic or ischemic stroke within the last three (3) months [b]
22. History of a cancer known to cause hemorrhagic metastases, e.g., melanoma, renal cell carcinoma, choriocarcinoma, thyroid carcinoma, lung carcinoma, breast carcinoma, and hepatocellular carcinoma [b]
23. History of left atrial myxoma [b]
24. Evidence of dissection in the intracranial cerebral arteries [b]
25. Significant mass effect with midline shift [b]
26. Patient is in a coma [b]

a. The exclusion criteria 12 was added to the protocol in December 2018.
b. The exclusion criteria 13-26 was added to the protocol in December 2021.

© 2023 Bahr-Hosseini M et al. *JAMA Network Open.*

eTable 2. Reasons for patients' ineligibility for intravenous thrombolysis and endovascular thrombectomy

Patient #	Reason for Exclusion from Lytic	Reason for Exclusion from EVT
1	Outside of window	Symptoms were deemed mild plus family opted not to go for EVT
2	Outside of window	The occlusion was deemed too distal (P2-P3)
3	Outside of window	The occlusion was deemed too distal (M3-M4)
4	Outside of window	The occlusion was deemed too distal (P2-P3)
5	Outside of window	The occlusion was deemed too distal (M3-M4)
6	Outside of window	The occlusion was deemed too distal (distal M2)
7	Outside of window	The occlusion was deemed too distal (P1-P2)
8	Outside of window	Symptoms were deemed mild and occlusion too distal (M2-M3)
9	On Coumadin	The occlusion was deemed too distal (distal M2)
10	On DOAC	The occlusion was deemed too distal (M3-M4)

© 2023 Bahr-Hosseini M et al. *JAMA Network Open.*

eTable 3. Demographic and Clinical Patient Features at Baseline

	Active (N=7)	Sham (N=3)
Age (y) (mean) (±SD)	75 (±19.6)	77 (±12)
Race (white) % (N)	86 (6)	33 (1)
Sex female % (N)	70 (5)	33 (1)
PMH % (N)		
Atrial fibrillation	0 (0)	67 (2)
Hypertension	86 (6)	67 (2)
Hyperlipidemia	86 (6)	67 (2)
Diabetes	29 (2)	33 (1)
Prior Stroke	29 (2)	33 (1)
Pre-stroke modified Rankin Scale (mRS), median(IQR)	0 (0-0)	0 (0-3)
Baseline NIHSS, mean (±SD)<br>Median(IQR)	8 (±8.5)<br>6 (2-10)	7(±2.6)<br>8(4-9)
Baseline Infarct Core Vol ml, median (IQR)	5 (1-26)	5.5 (2.75-5.75)
Baseline Perfusion Vol ml, median (IQR)	28 (15-33)	6 (5-7.5)
Baseline Penumbral Vol ml, median (IQR)	11 (0-23)	3 (1.75-3.5)
Baseline Hypoperfusion Intensity Ratio, median (IQR)	0.4 (0-0.6)	0 (0-0)
Baseline relative CBV index, median (IQR)	0.65 (0.6-0.9)	0.7 (0.6-0.7)
Location of vessel occlusion		
ICA % (N)	0 (0)	0 (0)
MCA-M1 % (N)	0 (0)	0 (0)
MCA-M2 % (N)	43 (3)	33 (1)
MCA- M3/M4 % (N)	14 (1)	67 (2)
PCA-P1 % (N)	14 (1)	0 (0)
PCA-P2/P3 % (N)	29 (2)	0 (0)
ACA-A1 % (N)	0 (0)	0 (0)
ACA-A2/A3 % (N)	0 (0)	0 (0)
PICA/AICA % (N)	0 (0)	0 (0)
Time from last known well time to tDCS initiation (hr), median (IQR)	7.7 (7-19.7)	14.4 (12.4-14.4)
Time from baseline MRI to tDCS initiation (hr), median (IQR)	2 (1.4-2.2)	1.9 (1.5-2.1)

© 2023 Bahr-Hosseini M et al. *JAMA Network Open.*

eTable 4. Main Outcomes Analysis

Feasibility Outcomes	Combined Active + Sham (n=10)	
tDCS Setup time (randomization to stimulation start) (min), median (IQR)	15 min (IQR 10-19)	
tDCS Setup time (randomization to stimulation start) in the last 4 enrolled patients (min),median (IQR)	12.5 min (IQR 9-15)	
Secondary Outcomes	**Active (N=7)**	**Sham (N=3)**
**Tolerability Outcomes**		
Rate of patients completing the protocol assigned stimulation % (N)	100 (7)	100 (3)
Rate of patients with intolerability resulting in a temporary stimulation cessation % (N)[a]	14 (1)	0 (0)
Rate of stimulation-related cutaneous, neurologic, nociceptive adverse effects % (N)	14 (1)	0 (0)
Nociceptive adverse effects % (N)	14 (1)	0 (0)
Cutaneous adverse effects % (N)	0 (0)	0 (0)
Neurological adverse effects % (N)	0 (0)	0 (0)
**Safety Outcome**		
Symptomatic Intracranial Hemorrhage % (N)[b]	14 (1)	0 (0)
Adverse Events		
Nonserious adverse events % (N)	14 (1)	0 (0)
Serious adverse events(SAE) % (N)	43 (3)	67 (2)
Unexpected SAE	0 (0)	0 (0)
SAE adjudicated as related to study treatment	0 (0)	0 (0)
Neurological deterioration within 24 hours % (N)	14 (1)	0 (0)
Mortality (mRS=6 at 90 days) % (N)	29 (2)	33 (1)

[a] In only one active patient, tDCS was briefly paused due to scalp burning sensation.
[b] One SICH occurred at Tier 2 of the study due to a ruptured mycotic aneurysm causing subarachnoid hemorrhage for which Tier 2 of the study was expanded to enroll 4 more patients.

© 2023 Bahr-Hosseini M et al. *JAMA Network Open.*

eTable 5. Per Protocol Exploratory Imaging Analysis

	**Active (N=5)[a]**	**Sham(N=3)**
Early Infarct core vol change ml , median (IQR)	+3 (0.5-12.5)	+8.7 (4.35-9.35)
Overall core vol change ml, median (IQR)	+3 (3-32)	+10 (6.5-10.25)
Early penumbral non-progression to core ml, median (IQR)	13 (2-20)	0 (0-0)
Early penumbral non-progression to core proportion, median (IQR)	74% (29-96.5)	0% (0-50)
Final penumbral salvage ml, median (IQR)	12 (2-17.5)	0 (0-1.5)
Final penumbral salvage proportion, median (IQR)	66% (29-80.5)	0% (0-0)
Early hypoperfusion lesion vol change ml, median (IQR)	-11 (-25 to -7)	0 (-2 to 12)
Early hypoperfusion lesion proportion change, median (IQR)	-33% (-84 to -31)	0% (-22 to +250)
Final hypoperfusion lesion vol change ml, median (IQR)	-28 (-32 to -7.5)	+9 (0 to 16.5)
Final hypoperfusion lesion proportion change, median (IQR)	-100% (-100 to -46)	+325% (+112 to +412)
Early quantitative rCBV change , median (IQR)	+64% (+40% to +110%)	-4% (-7 to +1)
Early Recanalization rate (N/total N)	80% (4/5)	33% (1/3)
Partial (N/total N)	40% (2/5)	33% (1/3)
Complete (N/total N)	40% (2/5)	0% (0)
Final Recanalization rate (N/total N)	80% (4/5)	33% (1/3)
Partial (N/total N)	40% (2/5)	33% (1/3)
Complete (N/total N)	40% (2/5)	0% (0)
Hemorrhagic transformation		
Hemorrhagic Infarction type 1	60% (3)[b]	0% (0)
Hemorrhagic Infarction type 2	0% (0)	33% (1)
Parenchymal Hemorrhage type 1	0% (0)	0% (0)
Parenchymal Hemorrhage type 2	0% (0)	0% (0)
Remote Intraparenchymal Hemorrhage	0% (0)	0% (0)
Subarachnoid Hemorrhage	0% (0)	0% (0)
Intraventricular Hemorrhage	0% (0)	0% (0)

[a] The exploratory per protocol analysis of imaging endpoints excluded 2 active group patients with protocol deviations: no penumbra was present at baseline on imaging core review (n=1) and septic embolization as stroke cause (n=1).
[b] One patient had HI1 at baseline imaging.

© 2023 Bahr-Hosseini M et al. *JAMA Network Open.*

eTable 6. Per Protocol Exploratory Clinical Analysis

**Exploratory Clinical Efficacy (Per Protocol Analysis)**	Active (N=5) [a]	Sham (N=3)
2-hour NIHSS change median(IQR)	0 (-3 to 0)	0 (-0.5 to +0.5)
24--hour NIHSS change, median (IQR)	-2 (-4 to 0.5)	-2 (-2.5 to -1)
90-day mRS 0-2 (%) (N)	60 (3)	67 (2)
90-day mRS 3-4 (%) (N)	40 (2)	0 (0)
90-day mRS 5-6 (%) (N)	0 (0)	33 (1)
90-day mRS		
0	0	0
1	2	1
2	1	1
3	2	0
4	0	0
5	0	0
6	0	1
90-day Barthel, median (IQR)	95 (87.5-97.5)	95 (47.5-97.5)

[a] The exploratory per protocol analysis of clinical endpoints excluded 2 active group patients with protocol deviations: no penumbra was present at baseline on imaging core review (n=1) and septic embolization as stroke cause (n=1).

© 2023 Bahr-Hosseini M et al. *JAMA Network Open.*
