# Supplementary material for: High-definition Cathodal Direct Current Stimulation for Treatment of Acute Ischemic Stroke: A Randomized Clinical Trial
Source: JAMA Netw Open. 2023 Jun 21;6(6):e2319231. doi: 10.1001/jamanetworkopen.2023.19231 (PMC10285579; doi:10.1001/jamanetworkopen.2023.19231)
Supplement: Supplement 3. — Nonauthor Collaborators [file jamanetwopen-e2319231-s003.pdf]

Supplemental Online Content: Nonauthor Collaborators
*First name, last name, and suffix (if applicable) are required and will appear in PubMed.

*Group Name(s): TESSERACT Trial Group							
***First Name and Middle Initial(s)**	***Last Name**	***Suffix (eg, Jr, III)**	Academic Degrees	Institution	Location (city, state/province, country)	Role or Contribution, eg, chair, principal investigator	Group (if more than 1 Group listed in the byline) and/or Subgroup (eg, Steering Committee)
Nerses	Sanossian		MD	Universty of Southern California	Los Angeles, CA	DSMB member	DSMB
Allan	Wu		MD	Northwestern University	Chicago, IL	DSMB member	DSMB
Bruce	Dobkin		MD	Universty of California Los Angeles	Los Angeles, CA	DSMB member	DSMB
Gilda	Avila		BS	Universty of California Los Angeles	Los Angeles, CA	Study Coordinator	Blinded Outcome Assessor
