# Supplementary material for: High-definition Cathodal Direct Current Stimulation for Treatment of Acute Ischemic Stroke: A Randomized Clinical Trial
Source: JAMA Netw Open. 2023 Jun 21;6(6):e2319231. doi: 10.1001/jamanetworkopen.2023.19231 (PMC10285579; doi:10.1001/jamanetworkopen.2023.19231)
Supplement: Supplement 4. — Data Sharing Statement [file jamanetwopen-e2319231-s004.pdf]

Data Sharing Statement

Bahr-Hosseini. High-Definition Cathodal Direct Current Stimulation for Treatment of Acute Ischemic Stroke. *JAMA Netw Open*. Published June 21, 2023.
doi:10.1001/jamanetworkopen.2023.19231

Data
Data available: Yes
Data types: Deidentified participant data
How to access data: https://clinicaltrials.gov/ct2/show/NCT03574038
When available: With publication

Supporting Documents
Document types: None

Additional Information
Who can access the data: NA
Types of analyses: NA
Mechanisms of data availability: NA
